# Engineering vascular potassium transport increases yield and drought resilience of cassava

**DOI:** 10.1038/s41477-025-02159-7

**Published:** 2025-12-17

**Authors:** W. Zierer, M. Fritzler, T. J. Chiu, R. B. Anjanappa, S.-H. Chang, R. Metzner, J. Quiros, C. E. Lamm, M. Thieme, R. Koller, G. Huber, O. Muller, U. Rascher, U. Sonnewald, H. E. Neuhaus, W. Gruissem, L. Bellin

**Affiliations:** 1 Division of Biochemistry, Department of Biology, Friedrich-Alexander-University Erlangen-Nuremberg, Erlangen, Germany; 2 Division of Plant Physiology, Department of Biology, University of Kaiserslautern-Landau (RPTU), Kaiserslautern, Germany; 3 Advanced Plant and Food Crop Biotechnology Center, National Chung Hsing University, Taichung, Taiwan; 4 Plant Biotechnology, Department of Biology, Eidgenössische Technische Hochschule (ETH) Zurich, Zurich, Switzerland; 5 Institute of Bio- and Geosciences: Plant Sciences (IBG-2), Forschungszentrum Jülich GmbH, Jülich, Germany; 6 Institute of Crop Science and Resource Conservation, Faculty of Agricultural, Nutritional and Engineering Sciences, University of Bonn, Bonn, Germany

**Keywords:** Plant molecular biology, Molecular engineering in plants, Plant physiology

## Abstract

Cassava (*Manihot esculenta*) is an important crop for food security in the tropics, particularly for smallholder farmers in sub-Saharan Africa, where yields are often severely limited by pathogen pressure, nutrient deficiency and water scarcity. We expressed a non-rectifying *Arabidopsis thaliana* potassium (K^+^) channel gene version, *AKT2*_*var*_, in the vascular tissue of cassava plants. The transgenic cassava plants had higher electron transport and CO_2_ assimilation rates, a higher bulk flow velocity and increased source–sink carbohydrate transport, as demonstrated by comparative ^11^C-positron emission tomography and tissue-specific metabolite profiling. Cassava storage root yield was significantly increased in greenhouse experiments and in a multi-year field trial conducted under subtropical conditions. *AKT2*_*var*_ plants were also more tolerant of drought stress and had higher storage root yield. Targeted alteration of K^+^ transport is therefore a promising strategy to improve cassava productivity without additional fertilizer input and in climate-adverse growing conditions.

## Abstract (web summary)

Zierer et al. engineered cassava to express a modified potassium channel that enhances sugar flow, improving the yield and drought resilience. This strategy offers a route to increase cassava productivity in tropical regions.

## Main

Cassava (*Manihot esculenta*) is native to the Amazon region and has become an important staple crop, feeding approximately 800 million people worldwide^1^. It is particularly important in sub-Saharan Africa as a major source of food security and economic support for smallholder farmers. Cassava can grow in poor soils and challenging environmental conditions, but storage root yields are well below its agronomic potential because smallholder farmers in sub-Saharan Africa often lack the financial means for fertilizer and irrigation^1,2^. Rising temperatures and unpredictable rainfall, leading to more extreme weather events such as droughts or floods, are also negatively affecting cassava production^3^. Innovative breeding and biotechnology strategies are urgently needed to secure and improve cassava storage root yields in sub-Saharan Africa without additional input.

Potassium (K^+^) is an essential macronutrient and a major determinant of crop yield^4,5^. In cassava, storage root yield is closely linked to the K^+^ status of the plant^6,7^. Earlier studies established a connection between K^+^ application and phloem transport^8–10^. Recent reports have confirmed the relationship between K^+^ and assimilate (sucrose) transport^11–13^. We argue that targeting K^+^ homeostasis can increase cassava phloem sucrose transport to storage roots and improve yield. Phloem transport relies on a solute concentration gradient to generate the necessary pressure to drive mass flow from source to sink tissues^14,15^. Although sugars, and particularly sucrose, are the major phloem osmolytes, K^+^ is the most abundant cation in the vasculature and is increasingly recognized as a contributor to phloem mass flow. Phloem K^+^ channels and transporters have been identified^12,13,16–18^, and K^+^ facilitates sucrose loading into the phloem^19^, most likely by energizing the phloem via a proton-motive force across the companion cell membrane^12^. The voltage-gated K^+^ channel AKT2 is specific to phloem companion cells^16^ and functions as an inward-rectifying K^+^ channel to load K^+^ into the companion cell, or, when phosphorylated, as a non-rectifying channel that both loads and releases K^+^ across the plasma membrane^19,20^. Circulating K^+^ energy stores, established by phosphorylated AKT2, could increase the efficiency of the H^+^-ATPase-dependent energization of transmembrane phloem loading^12,16^. The *Arabidopsis* AKT2(S210N,S329N) variant (here AKT2_var_) mirrors the function of the phosphorylated K^+^ channel and, when expressed in *Arabidopsis*, increases shoot growth, especially under energy-limiting conditions^12^.

**Fig. 1:**
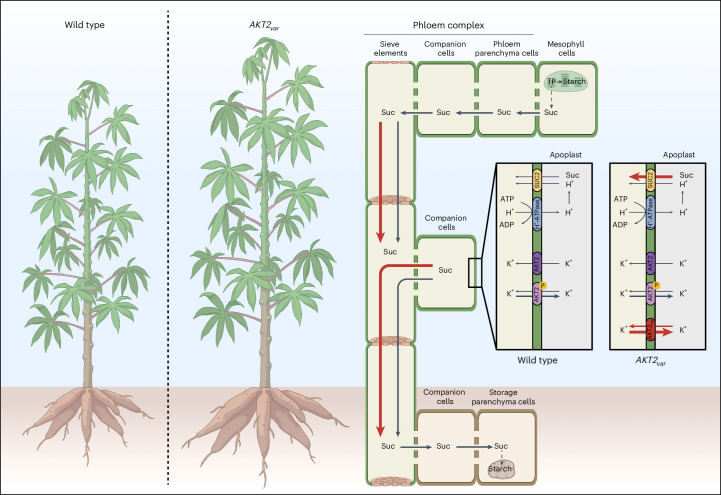
Model of the effects of the *AKT2*_*var*_ transgene in cassava, illustrating its role in increasing phloem transport rates. The black lines represent K^+^ and sucrose (Suc) flows in wild-type plants, and the red lines represent these flows in *AKT2*_*var*_ plants. In addition to endogenous AKT2, either phosphorylated or unphosphorylated, AKT2_var_ is introduced to modify phloem transport rates in the stem by altering K^+^ partitioning. This modification is predicted to facilitate sucrose allocation to storage roots by increasing phloem sucrose loading and reloading efficiency. TP, triose phosphate. Figure created with BioRender.com.

Although cassava is characterized by symplasmic phloem loading in leaves^21^ and symplasmic phloem unloading in storage roots^22^, the transport phloem still requires active sugar movement to retrieve leaked sucrose during long-distance transport (leakage-retrieval mechanism^23^). On the basis of the considerable transport distances of cassava and its high K^+^ demand for growth^6,7^, we propose that targeting K^+^ transport via vascular-tissue-specific expression of AKT2_var_ will facilitate sucrose allocation to growing storage roots, effectively increasing storage root yield (Fig. 1). Here, using non-invasive carbon tracer analysis, we demonstrate that cassava *AKT2*_*var*_ plants have a higher phloem transport velocity and improved agronomic performance in greenhouse and multi-year field experiments, particularly under water-limiting growth conditions.

## Results and discussion

### The *ArabidopsisAKT2* promoter drives vascular-tissue-specific expression of *AKT2*_*var*_ in cassava

**Fig. 2:**
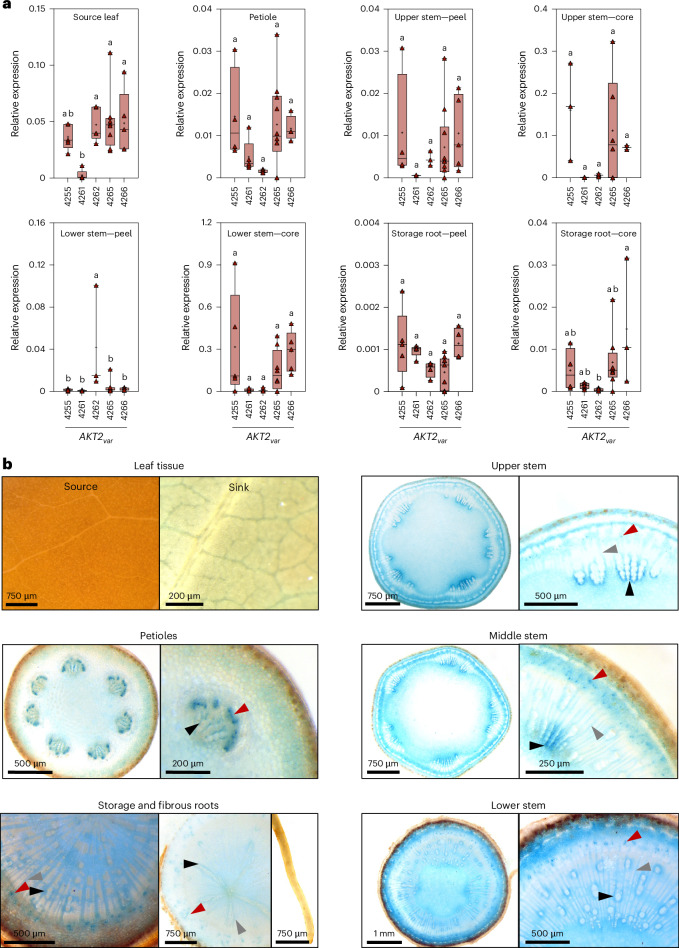
*ArabidopsisAKT2* promoter activity in cassava plants and vascular tissues. **a**, Quantitative real-time PCR measurements of relative *AKT2*_*var*_ mRNA expression levels in leaf; petiole; tuber; upper, middle and lower stem; and storage root after normalization to Me*GAPDH*. For *AKT2*_*va*r_-4255, *n* = 5, 4, 4, 3, 5, 5, 5 and 4; for *AKT2*_*va*r_-4261, *n* = 5, 5, 4, 3, 5, 5, 5 and 5; for *AKT2*_*va*r_-4262, *n* = 5, 5, 3, 3, 3, 3, 4 and 5; for *AKT2*_*va*r_-4265, *n* = 8, 10, 10, 5, 7, 8, 9 and 8; and for *AKT2*_*va*r_-4266, *n* = 5, 4, 5, 2, 5, 5, 4 and 3 biological replicates were analysed. In each box plot, the centre line represents the median, the plus sign indicates the mean, the box edges delineate the first and third quartiles, the whiskers extend to the maximum and minimum values and the triangles show individual values. Different lower-case letters indicate statistical significance, as calculated via one-way analysis of variance (ANOVA) with a post-hoc Tukey honestly significant difference (HSD) test (*P* < 0.05). **b**, Histochemical GUS staining patterns of representative *p*At*AKT2*::*GUS* transgenic cassava lines. Shown are source and sink leaves; cross-sections of petioles and upper, middle and lower stems; and the storage root and fibrous roots with root tip. Upper, middle and lower stem are defined as the green part of the stem near the apex (about 5 cm below the apex), the transition zone between the green stem and the brown/grey stem, and the base part of the stem, respectively. The red arrowheads mark phloem companion cells (dotted structures). The black arrowheads mark xylem parenchyma cells closely associated with xylem vessels. The grey arrowheads mark xylem ray cells connecting phloem and xylem. Source data

**Extended Data Fig. 1:**
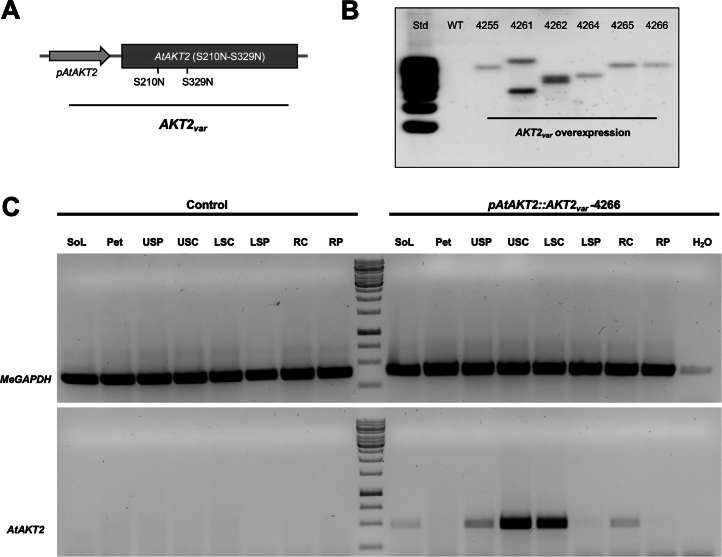
Characterization of T-DNA integration and additional expression information on *AKT2*_*var*_ plant lines. (**A**) Schematic representation of the mutant *AtAKT2* gene (S210N-S329N, *AKT2*_*var*_) fused to the Arabidopsis *AKT2* promoter that was transformed into cassava. (**B**) Southern Blot analysis of *AKT2*_*var*_ transgenic lines (*AKT2*_*var*_-4255, 4261, 4262, 4264, 4265 and 4266) compared to a wild type (WT) plant. (**C**) Semi-quantitative real-time PCR measurements of relative *AKT2*_*var*_ mRNA expression levels in source leaf tissue (SoL), petiole (Pet), upper stem peel (USP) and core (USC), lower stem peel (LSP) and core (LSC), and root peel (RP) and core (RC) with the corresponding *MeGAPDH* loading control of *AKT2*_*var*_-4266. The PCR was performed with 35 cycles. (**B-C**) Molecular marker is Gene Ruler 1 kb DNA Ladder (Thermo Fischer Scientific, Waltham, Massachusetts, USA). Primers “AKT2_CDS_RT_F1/R1” were used (see Methods section; ‘RT–PCR analysis’). Source data

We generated independent transgenic lines expressing the *AKT2*_*var*_ gene under the control of the *Arabidopsis* *AKT2* promoter in the cassava cultivar TMS60444 (Extended Data Fig. 1a). Four lines had a single transfer DNA insertion each (*AKT2*_*var*_-4255, -4264, -4265 and -4266), and two lines had two transfer DNA insertions (*AKT2*_*var*_-4261 and -4262) (Extended Data Fig. 1b). Quantitative PCR with reverse transcription (RT–qPCR) analysis using *AKT2*_*var*_-specific primers revealed a range of mRNA expression levels for *AKT2*_*var*_ in the transgenic lines, with the highest levels in the core tissue of the upper and lower stems (Fig. 2a). Transgenic cassava plants containing the *β-glucuronidase* (*GUS*) reporter gene under the control of the *Arabidopsis* *AKT2* promoter (*p*At*AKT2*::*GUS*) showed a GUS expression pattern generally consistent with the mRNA expression data (Fig. 2b). While the activity of the *AKT2* promoter in cassava was not entirely vascular specific, the strongest staining was observed in vascular-related cell types, particularly the phloem companion cells and vessel-associated parenchyma cells, and less staining in the connecting vascular rays (Fig. 2b). We independently confirmed the RT–qPCR and GUS staining results via end-point PCR with reverse transcription (RT–PCR) using *AKT2*_*var*_-4266 as an example. As expected, *AKT2*_*var*_ expression was strongest in the different cell types of the stem vasculature, with weaker expression in the vasculature of other plant parts (Extended Data Fig. 1c). The endogenous Me*AKT2a* and Me*AKT2b* genes are expressed in all cassava tissues analysed (Supplementary Fig. 1a–d), but Me*AKT2a* expression is highest in the phloem tissue, suggesting that MeAKT2a may be functionally equivalent to AtAKT2.

### AKT2_var_ increases phloem transport velocity, facilitating cassava growth and carbohydrate production

**Fig. 3:**
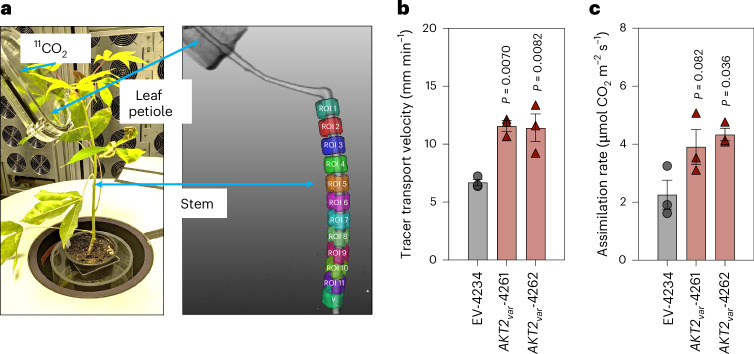
*AKT2*_*var*_ expression increases phloem tracer transport velocities and CO_2_ assimilation rates in cassava. **a**, Example of the experimental set-up for the analysis using ^11^C labelling and PET scanning. The coloured ROIs are used for the velocity analysis. **b**, Phloem flow velocities in *AKT2*_*var*_-4261 and *AKT2*_*var*_-4262 compared with EV-4234 plants. **c**, Altered CO_2_ assimilation rates of ^11^CO_2_-labelled leaves 12 weeks after planting in the greenhouse. The data in **b** and **c** are shown as the mean of three biological replicates plus or minus the standard deviation. Statistical significance was determined via one-way ANOVA with a post-hoc Tukey HSD test (*P* < 0.05); the *P* values are shown. Source data

Computational modelling has suggested that *AKT2*_*var*_ expression in *Arabidopsis* promotes the reloading of sucrose leaked from the phloem tissue^12^, which should lead to higher sucrose concentrations in the sieve elements and companion cells^12^ and consequently to higher phloem flow velocity, as proposed nearly 100 years ago^15^. However, the model prediction has not been experimentally verified in *Arabidopsis* or tested in crop plants. We therefore used positron emission tomography (PET) to measure phloem flow velocities along the cassava stem in empty vector (EV) control and *AKT2*_*var*_ lines. Stems were positioned inside the field of vision in a PET device, and 3D, time-resolved data were collected on a pulse-labelled ^11^CO_2_ tracer flowing from a leaf into the stem. The signal from the passing ^11^CO_2_ tracer pulse in subsequent stem sections inside the PET field of vision (see each coloured region of interest (ROI) on the stem in Fig. 3a) was used to calculate phloem flow velocities (Fig. 3b). *AKT2*_*var*_-4261 and *AKT2*_*var*_-4262 had significantly higher tracer transport velocities of 11.6 mm min^−1^ (74.5%) and 11.4 mm min^−1^ (70.7%), respectively, compared with 6.7 mm min^−1^ in EV-4234 (Fig. 3b). Photosynthetic fixation capacity was higher in *AKT2*_*var*_ plants, with CO_2_ assimilation rates of 3.9 µmol m^−2^ s^−1^ (77.3%) in *AKT2*_*var*_-4261 and 4.4 µmol m^−2^ s^−1^ (100%) in *AKT2*_*var*_-4262 compared with 2.2 µmol m^−2^ s^−1^ in EV-4234 plants (Fig. 3c).

**Extended Data Fig. 2:**
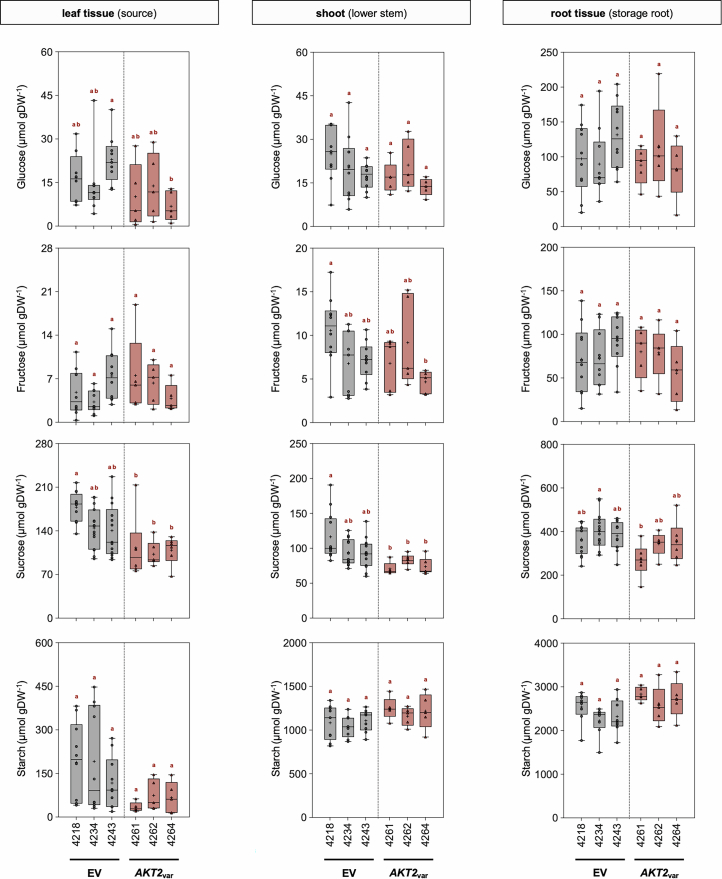
Expression of *AtAKT2*_*var*_ in cassava causes only minor changes in sugar and starch levels in greenhouse experiments. Glucose, fructose, sucrose, and starch concentrations in leaf, lower stem, and storage root tissues of plants from EV control lines (EV-4218, 4234, and 4243), and plants from *AKT2*_*var*_ lines (*AKT2*_*var*_-4261, 4262, and 4264) 19 weeks after planting in soil. From left to right for EV-4218 n = 10,10,9,8,8,10,8,8,8, EV-4234 n = 10,10,9,8,8,10,8,8,8, EV-4243 n = 10,10,10,8,8,10,8,8,8, *AKT2*_*var*_-4261 n = 5,5,5,4,5,5,5,4,5, *AKT2*_*var*_-4262 n = 5,5,5,5,5,5,5,4,5 and *AKT2*_*var*_-4264 n = 5,5,5,5,5,5,5,5,5 biological replicates were analysed. In box plots, centre line represents the median and plus (+) the mean, box edges delineate first and third quartiles, whiskers extend to maximum and minimum values and dots show individual values. Different lower-case letters indicate statistical significance, as calculated by one-way ANOVA with a post-hoc Tukey HSD test (*p* < 0.05). Source data

The increased transport velocity and assimilation rate in *AKT2*_*var*_ plants had no significant effect on ion (K^+^, Ca^2+^, Mg^2+^, PO_4_^−^ and SO_4_^2−^), glucose and fructose concentrations in source (leaf) and sink (stem and root) organs (Supplementary Figs. 2 and Extended Data Fig. 2), whereas sucrose and starch concentrations were markedly different (Extended Data Fig. 2). Leaf sucrose concentrations were reduced by 35% in *AKT2*_*var*_ compared with EV plants, and a comparable 30% reduction in sucrose levels was also measured in the lower stems of *AKT2*_*var*_ plants compared with EV lower stems. Since both the phloem transport velocity (Fig. 3b) and the photosynthetic rate (Fig. 3c) were higher in *AKT2*_*var*_ plants, the reduced shoot sucrose would be consistent with a facilitated source-to-sink transfer of assimilates. As a result of AKT2_var_ activity, tissue-specific starch concentrations were also altered. Although the differences were not significant due to high variation between the greenhouse samples, we found that starch levels were consistently reduced in *AKT2*_*var*_ leaves and increased in stems and storage roots (Extended Data Fig. 2).

**Fig. 4:**
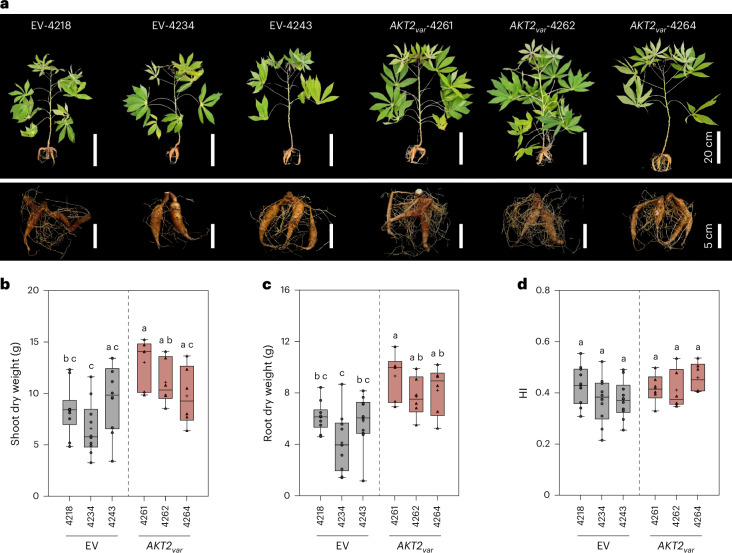
*AKT2*_*var*_ expression results in increased cassava growth rates under controlled greenhouse conditions. **a**, Representative phenotypes of shoot and root tissues from three vector control (EV-4218, EV-4234 and EV-4243) and three *AKT2*_*var*_ lines (*AKT2*_*var*_-4261, *AKT2*_*var*_-4262 and *AKT2*_*va*_-4264) are shown 19 weeks after planting. **b**–**d**, Shoot dry weight (**b**), storage root dry weight (**c**) and HI of dry weight (**d**). The data in **b**–**d** are shown as the mean of ten biological replicates for EV-4218, EV-4234 and EV-4243 and six biological replicates for *AKT2*_*va*r_-4261, *AKT2*_*var*_-4262 and *AKT2*_*var*_-4264 plus or minus the standard deviation. In each box plot, the centre line represents the median, the plus sign indicates the mean, the box edges delineate the first and third quartiles, the whiskers extend to the maximum and minimum values and the points show individual values. Different lower-case letters indicate statistical significance, as calculated via one-way ANOVA with a post-hoc Tukey HSD test (*P* < 0.05). Source data

Taken together, the data suggest that AKT2_var_ can increase long-distance bulk phloem transport of sucrose in cassava while maintaining ion homeostasis, with beneficial effects on carbohydrate accumulation in storage roots and stems. Agronomic evaluation of 19-week-old greenhouse-grown plants of *AKT2*_*var*_ and EV lines showed clear differences in growth performance in four independent cultivation experiments (Fig. 4 and Supplementary Fig. 3a–c). All *AKT2*_*var*_ plants showed an increase in total dry matter production compared with EV plants. Shoot dry weight was increased by 34.0%, 21.2% and 24.6% for *AKT2*_*var*_-4261, 4262 and 4264 plants, respectively, compared with EV plants (Fig. 4a,b).

Although cassava storage root development is still at an early stage at 19 weeks, *AKT2*_*var*_ plants already had a more than 55% higher storage root dry weight than EV plants (Fig. 4c), which at this growth stage did not affect harvest index (HI) (Fig. 4d).

### AKT2_var_ improves agronomic performance of field-grown cassava

**Extended Data Fig. 3:**
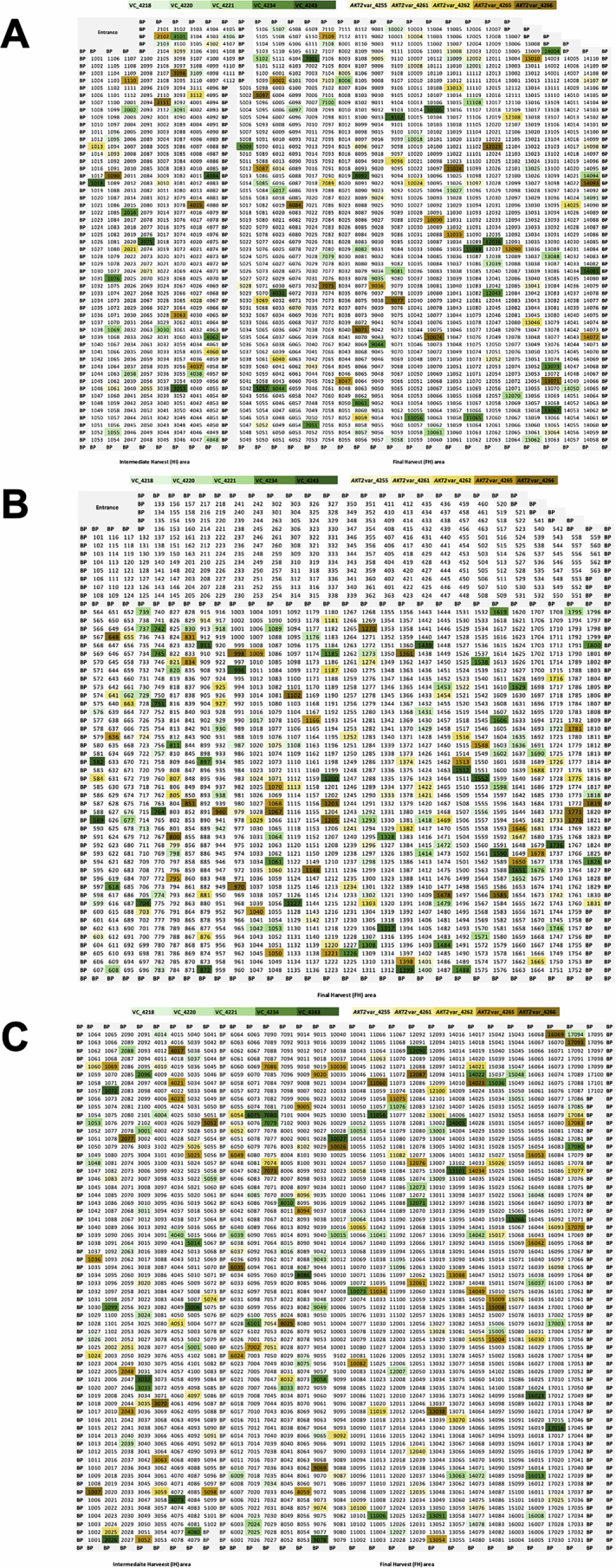
Serpentine randomization of planting for confined field trials in 2022, 2023, and 2024. Serpentine randomization of planting in (**A**) 2022, (**B**) 2023, and (**C**) 2024. Each four-digit number represents a plant grown in that year. Plants of EV lines are shown in shades of green and plants of *AKT2*_var_ lines are shown in shades of orange/brown. Four replications were grown for an intermediate harvest in 2022; 10 replications were grown for the final harvest in 2022. Seventeen replications were grown for the final harvest in 2023. Five replications were grown for an intermediate harvest in 2024; 12 replications were grown for the final harvest in 2024. Source data

While the data from cassava grown in the greenhouse indicate the positive effect of *AKT2*_*var*_ on plant growth and storage root yield in line with the model assumptions (Fig. 1), they cannot predict the agronomic performance of the *AKT2*_*var*_ plants in the field. We therefore grew plants from the *AKT2*_*var*_-4255, -4261, -4262, -4265 and -4266 lines together with plants from the EV-4218, -4220, -4221, -4234 and -4243 control lines in three highly replicated randomized field trials (Extended Data Fig. 3) under subtropical conditions during 2022–2024.

**Fig. 5:**
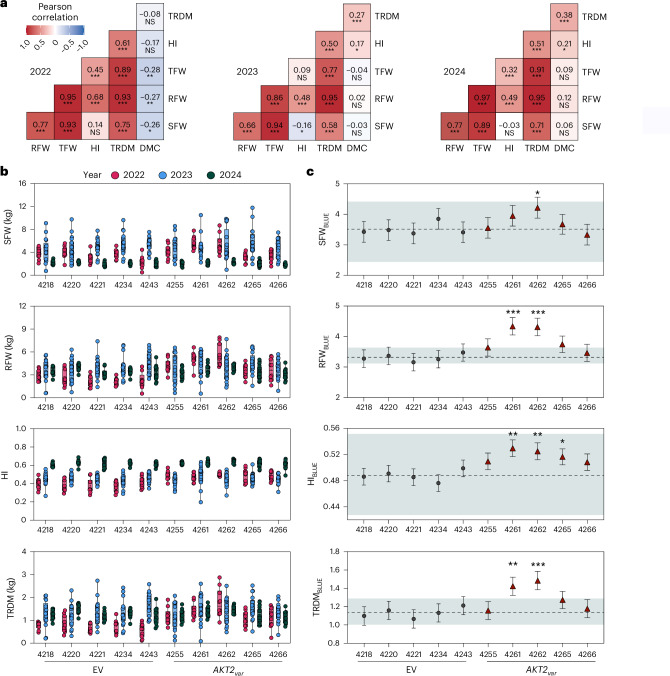
Analysis of multi-year agronomic performance of *AKT2*_*var*_ and EV control plants in field trials. **a**, Pearson correlation of agronomic traits per year using data from both *AKT2*_*var*_ and EV plants. Positive and negative correlations are shown in red and blue, respectively. Significance was tested using a two-sided Student’s *t*-test. TFW, total fresh weight; DMC, dry matter content. **b**, Line performance for each year. Spatially corrected data values of replicates (at least ten) are shown for years and traits. **c**, Line performance after spatial and temporal correction of the raw data (Methods). Adjusted means (BLUEs) ± s.e. of genotype effects for each line and trait are shown. The values represent BLUEs obtained from a linear mixed-effects model of spatially corrected trait values, with line fitted as a fixed effect and year and replicate as random effects (trait spatially corrected ~ line + (1|year) + (1|replicate)). The error bars show the model-derived standard errors of the adjusted means. In each plot, the dashed horizontal line indicates the mean value of the reference line, and the shaded grey area represents its mean ± s.e. The asterisks denote significance levels as follows: not significant (NS, *P* > 0.05), significant (*P < 0.05), highly significant (**P < 0.01) and very highly significant (***P < 0.001). Source data

While absolute growth and storage root yield based on agronomic (Supplementary Figs. 4–6) and unmanned aerial vehicle (UAV) measurements (Supplementary Fig. 7) varied for both AKT2_var_ and EV plants between experiments, agronomic performance data confirmed a significant positive correlation between shoot and storage root biomass production (Fig. 5). The correlation was less pronounced in 2023 (*R* = 0.66) than in 2022 or 2024 (*R* = 0.77), but such variability is not unexpected in the field under varying environmental conditions (Supplementary Fig. 8). The relative storage root dry matter content of all plants in 2022 had a small negative but significant correlation with the fresh weight traits, which was not observed in 2023 or 2024 (Fig. 5a). However, *AKT2*_*var*_ plants still maintained high levels of total storage root dry matter (TRDM) in all three years (Fig. 5b). Spatial correction of the field data using SpATS^24^ in R and normalization to the mean of the EV plant data for each year allowed us to comparatively assess the performance of *AKT2*_*var*_ and EV plants across the independent field experiments and to calculate best linear unbiased estimates (BLUEs) for shoot fresh weight (SFW), root fresh weight (RFW), TRDM and HI (Fig. 5c). The multi-year statistical analysis confirmed a significant increase in SFW for *AKT2*_*var*_-4262 and significant increases in RFW and TRDM for *AKT2*_*var*_-4261 and -4262. HI was also consistently and significantly improved in *AKT2*_*var*_-4261, -4262 and -4265 compared with EV lines (Fig. 5c). As the growth period during the field trials was 8 months compared with the typical 10–12 months for harvestable TSM60444 storage roots, and the plants were grown from tissue culture plantlets rather than stakes, it is likely that the agronomic parameters of *AKT2*_*var*_ plants will continue to outperform those of EV plants at maturity.

**Extended Data Fig. 4:**
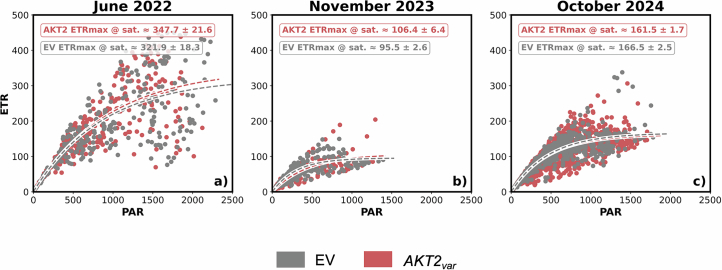
Summary of electron transport rate (ETR) data obtained during confined field trials. Recorded ETR values at different Photosynthetically Active Radiation (PAR) levels are shown for plants from EV and *AKT2*_*var*_ lines grown during confined field trials in 2022 to 2024. The dashed lines represent the integrated curves calculated from all values obtained from EV and *AKT2*_*var*_ plants. Data from 2022 was collected in June from approximately 3-month-old cassava plants, data from 2023 was collected in November from approximately 8-month-old cassava plants, and data from 2024 was collected in October from approximately 7-month-old cassava plants. Data collection is shown in Supplementary Fig. 13. Source data

**Extended Data Fig. 5:**
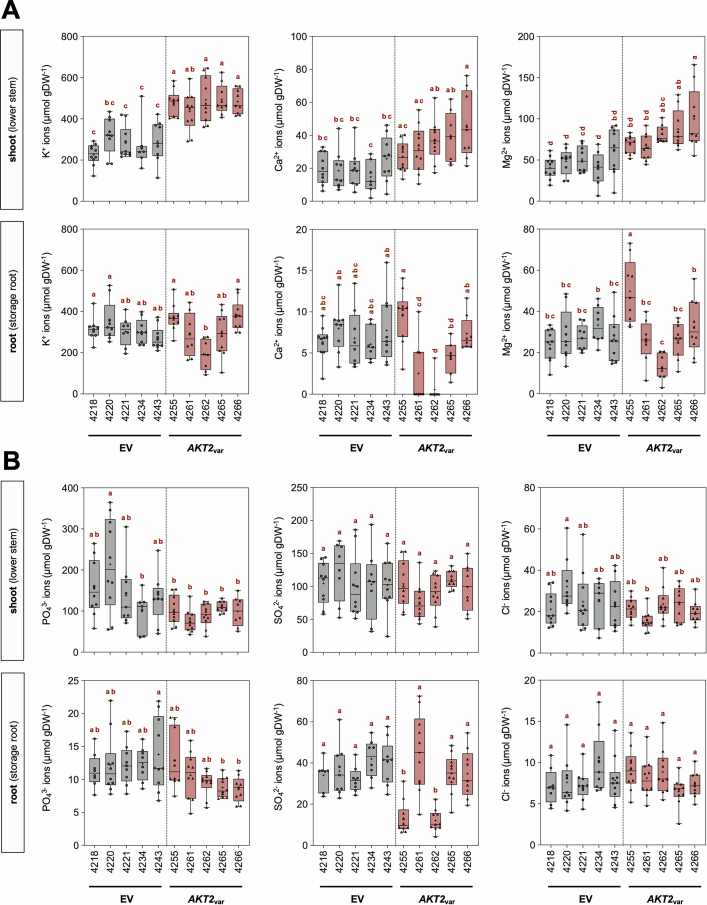
*AKT2*_*var*_ expression in cassava causes alterations in cation and anion concentrations in confined field trials in 2022. (**A**) Cation contents of potassium (K^+^), calcium (Ca^2+^), and magnesium (Mg^2+^) in lower stem and storage root tissues. From left to right for EV-4218 n = 10,10,10,9,10,10, EV-4220 n = 9,9,10,9,9,9, EV-4221 n = 10,10,10,8,8,9, EV-4234 n = 7,8,8,9,7,9, EV-4243 n = 9,9,10,10,9,10, *AKT2*_*var*_-4255 n = 10,10,10,10,10,9, *AKT2*_*var*_-4261 n = 10,10,10,8,8,8, *AKT2*_*var*_-4262 n = 10,10,9,8,8,8, *AKT2*_*var*_-4265 n = 9,8,9,10,7,10 and *AKT2*_*var*_-4266 n = 9,9,9,10,8,10 biological replicates were analysed. (**B**) Anion contents of phosphate (PO_4_^3−^), sulphate (SO_4_^2−^), and chloride (Cl^−^) in lower stem and storage root tissues. Data are shown for plants from EV lines (EV-4218, 4234, and 4243) and *AKT2*_*var*_ lines (*AKT2*_*var*_-4261, 4262, and 4264) that were harvested about nine months (April to December) after planting. From left to right for EV-4218 n = 10,10,10,8,7,8, EV-4220 n = 10,8,9,10,9,9 EV-4221 n = 9,10,10,9,8,9, EV-4234 n = 7,8,8,8,8,9, EV-4243 n = 9,10,9,10,9,10, *AKT2*_*var*_-4255 n = 10,10,10,10,10,10, *AKT2*_*var*_-4261 n = 10,10,10, 10,10,10, *AKT2*_*var*_-4262 n = 10,10,10, 10,10,10, *AKT2*_*var*_-4265 n = 9,9,9,10,10,10 and *AKT2*_*var*_-4266 n = 8,8,9,9,9,9 biological replicates were analysed. In box plots, centre line represents the median and plus (+) the mean, box edges delineate first and third quartiles, whiskers extend to maximum and minimum values and dots show individual values. Different lower-case letters indicate statistical significance, as calculated by one-way ANOVA with a post-hoc Tukey HSD test (*p* < 0.05). Source data

**Extended Data Fig. 6:**
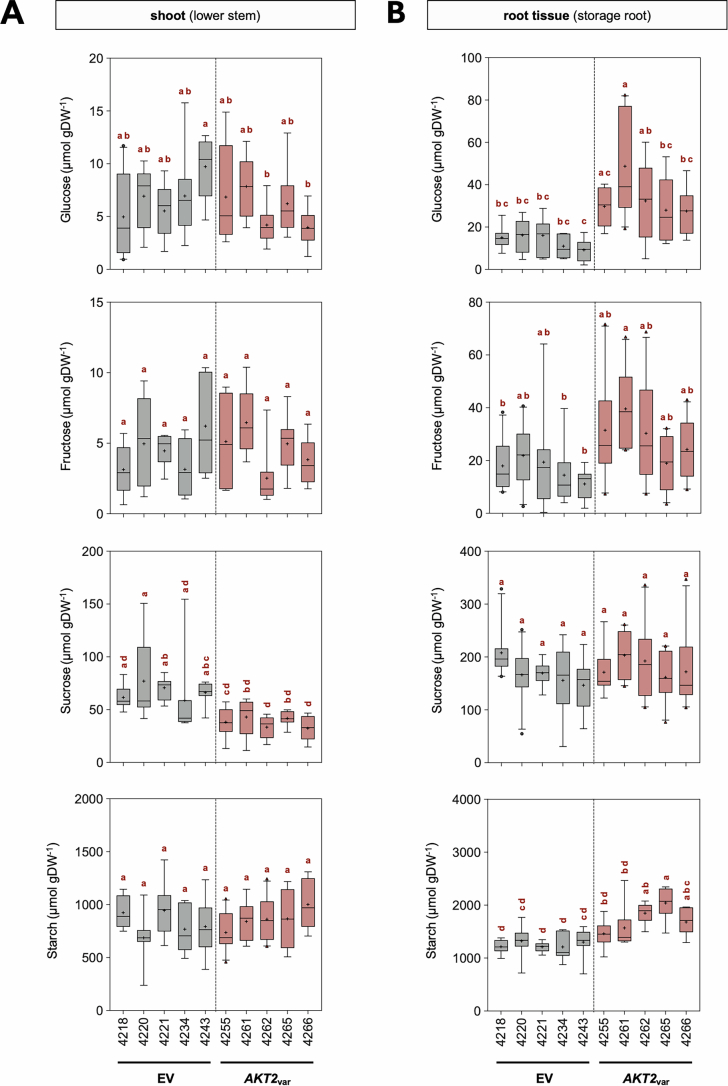
Expression of *AKT2*_*var*_ in cassava leads to changes in sugar concentrations in 2022 confined field trials. Glucose, fructose, sucrose, and starch concentrations in (**A**) shoot and (**B**) storage root tissues. Data are shown for plants from EV lines (EV-4218, EV-4234, and EV-4243) and *AKT2*_*var*_ lines (*AKT2*_*var*_-4261, *AKT2*_*var*_-4262, and *AKT2*_*var*_-4264) about nine months (April to December) after planting. In A for EV-4218 n = 10,8,9,9, EV-4220 n = 8,7,9,8, EV-4221 n = 9,7,8,9, EV-4234 n = 9,8,8,8, EV-4243 n = 8,8,9,9, *AKT2*_*var*_-4255 n = 8,6,9,10, *AKT2*_*var*_-4261 n = 8,5,8,9, *AKT2*_*var*_-4262 n = 9,8,9,10, *AKT2*_*var*_-4265 n = 9,8,8,9, and *AKT2*_*var*_-4266 n = 6,7,9,6 biological replicates were analysed.In B for EV-4218 n = 9,10,10,9, EV-4220 n = 8,10,10,9, EV-4221 n = 7,9,8,8, EV-4234 n = 7,7,9,7, EV-4243 n = 8,9,9,7, *AKT2*_*var*_-4255 n = 8,10,9,9, *AKT2*_*var*_-4261 n = 10,10,10,9, *AKT2*_*var*_-4262 n = 9,10,10,9, *AKT2*_*var*_-4265 n = 8,10,10,9, and *AKT2*_*var*_-4266 n = 8,10,10,9 biological replicates were analysed. In box plots, centre line represents the median and plus (+) the mean, box edges delineate first and third quartiles, whiskers extend to maximum and minimum values and dots show individual values. Different lower-case letters indicate statistical significance, as calculated by one-way ANOVA with a post-hoc Tukey HSD test (*p* < 0.05). Source data

We next asked whether the improved agronomic performance of *AKT2*_*var*_ field-grown plants was correlated with increased photosynthetic activity as well as sucrose and ion partitioning. The electron transport rate (ETR) is a key indicator of photosynthetic performance (that is, photosynthetic efficiency multiplied by irradiance, saturating at high irradiance as ETR_max_; ref. ^25^). ETR_max_ was 8% higher in *AKT2*_*var*_ plants than in EV plants under saturating light conditions in 2022, indicating that AKT2_var_ improves photosynthetic efficiency under field conditions at the early growth stage (Extended Data Fig. 4). In 2023 and 2024, at a later growth stage, *AKT2*_*var*_ and EV plants showed similar ETR irradiance responses (Extended Data Fig. 4). *AKT2*_*var*_ field-grown plants had an almost 70% higher K^+^ concentration in the lower part of the stem (Extended Data Fig. 5), which can explain the almost 40% reduced sucrose concentration (38.5 µmol per g dry weight) in the lower part of the stem of *AKT2*_*var*_ plants compared with that in EV plants (66.9 µmol per g dry weight) (Extended Data Fig. 6). This difference in K^+^ concentration was not detected in greenhouse plants (Supplementary Fig. 2), suggesting that AKT2_var_ alters K^+^ homeostasis later in cassava development or under specific environmental conditions. We observed a lower K^+^ concentration in the storage roots of *AKT2*_*var*_-4261 and -4262 plants, but this difference was not consistent in plants from all *AKT2*_*var*_ lines (Extended Data Fig. 5).

While other cations (Ca^2+^ and Mg^2+^) followed the K^+^ trend in *AKT2*_*var*_ plants, measured anion (Cl^−^, PO_4_^−^ and SO_4_^2−^) as well as glucose and fructose concentrations were not significantly different between *AKT2*_*var*_ and EV plants (Extended Data Figs. 5 and 6). In addition to the altered K^+^ homeostasis in *AKT2*_*var*_ plants, we found a positive effect on starch accumulation in storage roots, with starch concentrations >50% higher in the best-performing *AKT2*_*var*_ lines (Extended Data Fig. 6), consistent with the early increase in starch concentrations in the greenhouse plants (Extended Data Fig. 2).

We propose that AKT2_var_-driven K^+^ homeostasis facilitates bulk phloem transport of sucrose from source to sink tissues in the field-grown *AKT2*_*var*_ cassava plants, thereby increasing their starch production and storage root yield.

### *AKT2*_*var*_ plants are resistant to drought stress

**Fig. 6:**
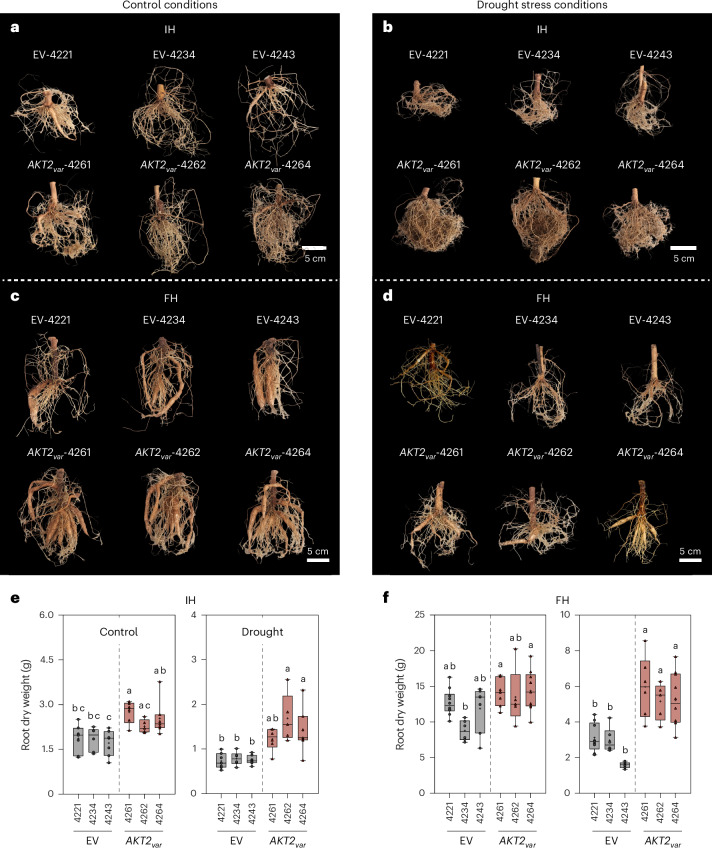
*AKT2*_*var*_ expression significantly improves resistance of cassava to drought stress. **a**–**f**, To induce drought stress in plants from EV and *AKT2*_*var*_ lines, all plants were first grown under controlled conditions in the greenhouse for 8 weeks. This was followed by either a 5-week period of drought stress with watering only once a week (drought stress condition) or a 5-week period of daily watering (control condition). After these 5 weeks, an IH was carried out for both conditions. Both conditions were then returned to the watering regime of the control condition for 5 weeks. The FH was carried out after this 5-week recovery period. Panels **a**–**d** show representative phenotypes of root tissue from three EV plants (EV-4221, EV-4234 and EV-4243) and three *AKT2*_*var*_ plants (*AKT2*_*var*_-4261, *AKT2*_*var*_-4262 and *AKT2*_*va*_-4264) at IH (**a**,**b**) and FH (**c**,**d**). Root dry weight was determined at IH (*n* = 7 biological replicates for EV-4221, *n* = 6 for EV-4234, *n* = 7 for EV-4243, *n* = 6 for *AKT2*_*var*_-4261, *n* = 5 for *AKT2*_*var*_-4262 and *n* = 7 for *AKT2*_*var*_-4264) (**e**) and FH (*n* = 10 and 9 biological replicates for EV-4221, *n* = 6 for EV-4234, *n* = 7 for EV-4243, *n* = 7 and 6 for *AKT2*_*var*_-4261, *n* = 5 for *AKT2*_*var*_-4262 and *n* = 10 for *AKT2*_*var*_-4264) (**f**) for both the control and drought conditions. Images were digitally extracted for comparison in **a**–**d**. In each box plot, the centre line represents the median, the plus sign indicates the mean, the box edges delineate the first and third quartiles, the whiskers extend to the maximum and minimum values and the points show individual values. Different lower-case letters indicate statistical significance, as calculated via one-way ANOVA with a post-hoc Tukey HSD test (*P* < 0.05). Source data

**Extended Data Fig. 7:**
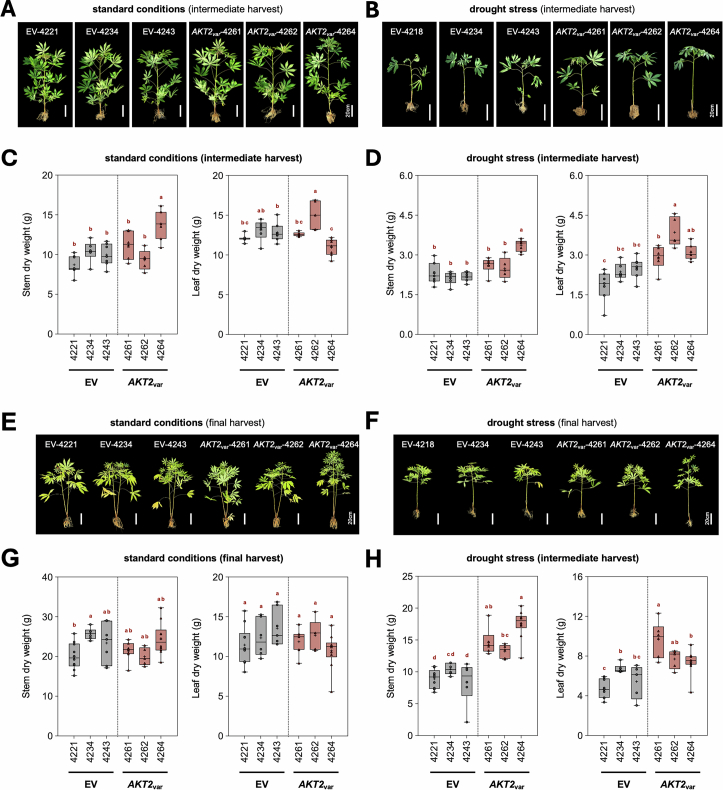
*AKT2*_*var*_ expression not only enhances cassava growth under standard conditions, but also significantly improves the response to drought stress. Plants from EV and *AKT2*_*var*_ lines were grown, treated for drought and sampled according to the schedule shown in Supplementary Fig. 9. Six biological replicates were analyzed per line. (**A, B**) Phenotypes of shoots and roots from plants of EV lines (EV-4221, EV-4234, and EV-4243) and *AKT2*_*var*_ lines (*AKT2*_*var*_-4261, *AKT2*_*var*_-4262, and *AKT2*_*var*_-4264) at intermediate harvest (IH). (**C, D**) Stem, leaf, and root dry weights were measured at intermediate harvest under either standard or drought stress conditions. For EV-4221 n = 7, EV-4234 n = 6, EV-4243 n = 7, *AKT2*_*var*_-4261 n = 6, *AKT2*_*var*_-4262 n = 5 and *AKT2*_*var*_-4264 n = 7 biological replicates were analysed. (**E, F**) Phenotypes of shoots and roots from plants of EV lines (EV-4221, 4234, and 4243), and *AKT2*_*var*_ lines (*AKT2*_*var*_-4261, 4262, and 4264) at final harvest (FH). (**G, H**) Stem, leaf, and root dry weights were measured at final harvest under either control, or after drought stress conditions. For EV-4221 n = 10,9 EV-4234 n = 6,6 EV-4243 n = 7,6 *AKT2*_*var*_-4261 n = 7,6, *AKT2*_*var*_-4262 n = 5,5 and *AKT2*_*var*_-4264 n = 10,10 biological replicates were analysed. In box plots, centre line represents the median and plus (+) the mean, box edges delineate first and third quartiles, whiskers extend to maximum and minimum values and dots show individual values. Different lower-case letters indicate statistical significance, as calculated by one-way ANOVA with a post-hoc Tukey HSD test (*p* < 0.05). Source data

**Extended Data Fig. 8:**
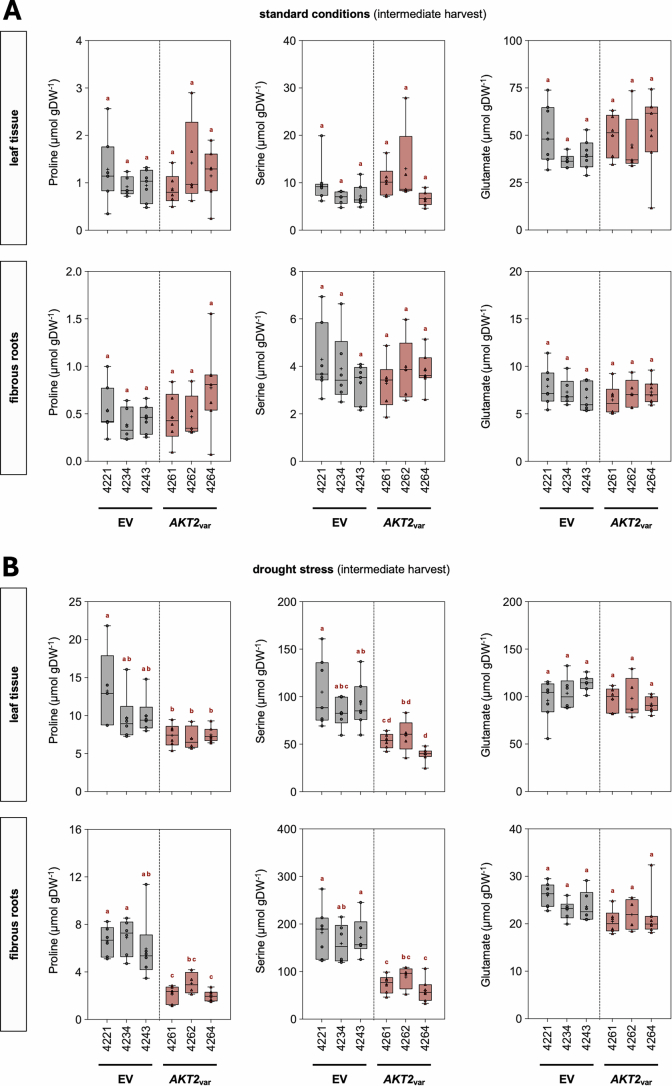
*AKT2*_*var*_ lines have reduced levels of stress-related amino acids during drought stress. Plants from EV and *AKT2*_*var*_ lines were grown, treated for drought and sampled according to the schedule shown in Supplementary Fig. 9. The amino acids proline, serine, and glutamate were measured in leaf tissue and fibrous roots of plants from all analysed EV lines (EV-4221, EV-4234, and EV-4243) and *AKT2*_*var*_ lines (*AKT2*_*var*_-4261, *AKT2*_*var*_-4262, and *AKT2*_*var*_-4264) under (**A**) control conditions (**B**) or during the drought stress phase at intermediate harvest. In A for EV-4221 n = 7, EV-4243 n = 6, EV-4243 n = 7, *AKT2*_var_-4261 n = 6, *AKT2*_var_-4262 n = 5 and *AKT2*_var_-4264 n = 7 biological replicates were analyzed. In B for EV-4221 n = 5,7,7,6,7,7, EV-4243 n = 6,6,6,6,6,6, EV-4243 n = 6,7,7,6,6,6, *AKT2*_var_-4261 n = 6,6,6,6,6,6, *AKT2*_var_-4262 n = 4,5,5,4,4,4 and *AKT2*_var_-4264 n = 7,7,7,7,7,7 biological replicates were analyzed. In box plots, centre line represents the median and plus (+) the mean, box edges delineate first and third quartiles, whiskers extend to maximum and minimum values and dots show individual values. Different lower-case letters indicate statistical significance, as calculated by one-way ANOVA with a post-hoc Tukey HSD test (*p* < 0.05). Source data

The variable precipitation pattern between the years (Supplementary Fig. 8) suggested that differences in the plant water status affect the agronomic performance of *AKT2*_*var*_ plants in the field. Water availability is closely linked to plant K^+^ homeostasis^26,27^, and increased carbon supply to sink organs may stimulate the growth of the fibrous root network. Also, the At*AKT2* promoter contains six ABRE motifs and has been shown to be induced by abscisic acid^28^. We therefore investigated the growth performance of *AKT2*_*var*_ plants under controlled drought conditions. We subjected 8-week-old *AKT2*_*var*_ and EV plants grown in the greenhouse to a 5-week drought treatment to assess their growth performance relative to control plants immediately after the drought treatment (intermediate harvest (IH) at week 13) or after a further 5 weeks of normal watering (final harvest (FH); Supplementary Fig. 9). Under continuous irrigation, *AKT2*_*var*_ and EV plants had similar root system morphology at IH and FH (Fig. 6 and Extended Data Fig. 7). As previously observed (Figs. 4 and 5), all plants from the *AKT2*_*var*_-4261, -4262 and -4264 lines produced almost 30% higher root biomass than EV plants (Fig. 5e,f). Importantly, at IH after drought stress and at FH after rewatering, *AKT2*_*var*_ plants had an increased storage root dry weight that was >100% higher at FH than in EV plants, indicating that *AKT2*_*var*_ plants had initiated and maintained their storage root growth during the drought period (Fig. 6e,f). Stem dry weight was also higher in drought-treated *AKT2*_*var*_ plants than in EV plants (Extended Data Fig. 7). The *AKT2*_*var*_ transgene expression also strongly increased in response to drought stress (Supplementary Fig. 10). Together, this suggests that AKT2_var_ has a beneficial physiological effect on cassava growth performance during drought. The levels of the amino acids serine and proline can be used as proxies for the drought status of plants^29^, whereas the levels of amino acids such as glutamine are typically not altered by this treatment. *AKT2*_*var*_ and EV plants had no significant differences in serine, proline and glutamine concentrations in leaves and fibrous roots under well-watered conditions. However, serine and proline concentrations specifically increased in EV plants at IH after the drought period, whereas glutamine concentrations remained unchanged (Extended Data Fig. 8). Although proline and serine concentrations also increased in *AKT2*_*var*_ plants at IH after the drought period, they remained significantly lower than those in EV plants (Extended Data Fig. 8), indicating that the physiological changes associated with drought stress were more moderate in *AKT2*_*var*_ plants.

**Extended Data Fig. 9:**
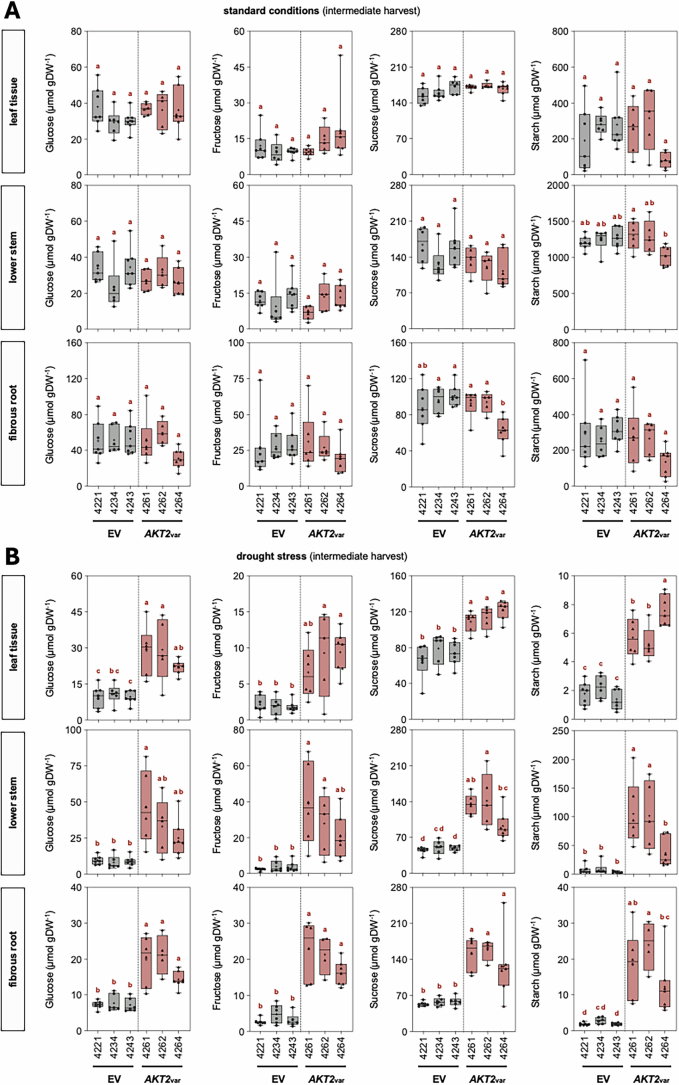
*AKT2*_*var*_ expression in cassava leads to increased carbohydrate levels during periodic drought stress. Glucose, fructose, sucrose, and starch concentrations in leaf, lower stem, and fibrous root tissues of plants from EV lines (EV-4221, 4234, and 4243) and *AKT2*_*var*_ lines (*AKT2*_*var*_-4261, 4262, and 4264) under (**A**) control conditions or (**B**) during periodic drought stress. In A for EV-4221 n = 7,7,7,7,7,7,6,7,7,7,7,7, EV-4243 n = 6,6,7,6,6,6,6,6,6,6,6,6, EV-4243 n = 7,7,6,7,7,7,7,7,7,7,7,7, *AKT2*_var_-4261 n = 6,6,6,6,6,6,5,6,6,6,6,6 *AKT2*_var_-4262 n = 5,5,5,5,5,5,5,5,5,5,5,5, and *AKT2*_var_-4264 n = 7,7,7,7,7,7,7,7,7,7,7,7 biological replicates were analyzed. In B for EV-4221 n = 7,7,6,7,7,7,7,7,7,7,7,7, EV-4243 n = 6,6,6,6,6,6,6,6,6,6,6,6, EV-4243 n = 7,7,7,7,7,7,6,7,6,6,6,7, *AKT2*_var_-4261 n = 6,6,6,6,6,6,6,6,6,6,6,6, *AKT2*_var_-4262 n = 5,5,5,5,5,4,4,4,5, and *AKT2*_var_-4264 n = 7,7,7,7,7,7,7,7,7,7,7,7, biological replicates were analyzed. In box plots, centre line represents the median and plus (+) the mean, box edges delineate first and third quartiles, whiskers extend to maximum and minimum values and dots show individual values. Different lower-case letters indicate statistical significance, as calculated by one-way ANOVA with a post-hoc Tukey HSD test (*p* < 0.05). Source data

The drought treatments of *AKT2*_*var*_ and EV plants had no significant effect on the concentrations of cations (Na^+^, K^+^, Ca^2+^ and Mg^2+^) and anions (Cl^−^, NO^3−^, PO_4_^3−^ and SO_4_^2−^) at IH (Supplementary Figs. 11 and 12). Sucrose, glucose and fructose concentrations were significantly increased in *AKT2*_*var*_ plant organs at IH after drought treatment (Extended Data Fig. 9b,d), especially sucrose concentrations, which were almost three times higher in stem and root tissues (Extended Data Fig. 9b). This resulted in up to 50% higher starch concentrations in the leaves and stems of *AKT2*_*var*_ plants than in those of EV plants (Extended Data Fig. 9b), suggesting that *AKT2*_*var*_ plants maintained high photosynthetic capacity and metabolism during drought treatment.

## Conclusions

As an essential macronutrient, K^+^ affects the plant water balance by controlling stomatal aperture, thereby affecting photosynthetic efficiency and transpiration rates^30,31^, and maintains cell turgor and osmotic balance during abiotic stress conditions^32^. The results of our study show that targeting K^+^ homeostasis with AKT2_var_ reproducibly increases phloem mass flow velocity, which improves the agronomic performance of cassava under greenhouse and field conditions and during drought stress. Phloem sucrose transport and K^+^ concentrations are closely linked, as higher phloem K^+^ concentrations can maintain phloem pressure and flow rate when sucrose is reduced in maize^11^, and phloem K^+^ loading and recirculation between shoots and roots affect carbon allocation and grain yield in rice^13^. Similar to *Arabidopsis*^33^, both maize and rice are apoplasmic sucrose loaders^34,35^ in which AKT2 orthologues maintain K^+^ homeostasis, and expression of AKT2_var_ channels may also improve their yield, but this has not been experimentally tested. Most importantly, although cassava is a symplasmic sucrose phloem loader^21^ in which the function of the AKT2 orthologues is not currently understood, our results show that cassava plants expressing the *Arabidopsis* AKT2_var_ channel have higher phloem transport rates and high photosynthetic efficiency, facilitating source-to-sink transport of sucrose and improving the agronomic performance and storage root yield of the crop even during water stress.

While the positive impact of AKT2_var_ on cassava agronomic performance is convincing, the underlying mechanism currently remains unresolved. It is possible that in cassava, AKT2_var_ establishes a “potassium battery” proposed for apoplasmic phloem loaders such as *Arabidopsis*^19^. In this scenario, AKT2_var_ would enhance K^+^ efflux from companion cells, which strengthens the proton motive force that drives sucrose uptake via H^+^-coupled transporters. Cassava is a symplasmic phloem loader^21^, however, and it is uncertain whether this model applies because assimilate retrieval along the transport pathway would not rely strongly on proton-coupled sucrose loading. Alternatively, AKT2_var_ could promote K^+^ circulation between the shoot and the root to amplify the longitudinal K^+^ gradient and increase the phloem pressure gradient, as proposed in other models^36,37^. Distinguishing between these possibilities will require further experiments.

AKT2_var_ is a promising molecular breeding tool for enhancing cassava productivity under both optimal and stress conditions. It can now be introduced into farmer-preferred and commercial cassava varieties for further testing and product development. Clarifying the precise mode of AKT2_var_ action will also be useful for future development of more productive cassava varieties and harnessing K^+^ dynamics for increasing the yield of other major agricultural crops.

## Methods

### Generation of DNA constructs

All binary vectors for genetic transformation were constructed using the Golden Gate cloning toolkit^38^. The *p*At*AKT2*::*AKT2*_*var*_ expression cassette contained the following DNA elements: the promoter and 5′ untranslated region (1,500 bp) of the *Arabidopsis thaliana POTASSIUM TRANSPORTER 2* gene (*p*At*AKT2*), the At*AKT2* gene coding sequence with S210N and S329N amino acid changes (*AKT2*_*var*_), an amino-terminal 6xHA-tag and the *Arabidopsis thaliana HEAT SHOCK PROTEIN 18.2* (*HSP18.2*) 3′ untranslated region. The *p*At*AKT2*::*GUS* expression cassette contained the following DNA elements: the promoter and 5′ untranslated region (1,500 bp) of At*AKT2*, the modified *GUS* (‘GUSPlus’^39^) gene coding sequence and the *Agrobacterium tumefaciens NOPALINE SYNTHASE* (Atu*NOS*) terminator. Both plasmids contained the following selectable DNA marker cassette: the Atu*NOS* promoter, the *HYGROMYCIN PHOSPHOTRANSFERASE* (*hpt2*) gene coding sequence and the *CAULIFLOWER MOSAIC VIRUS 35S* terminator. The plasmid sequences are provided in Supplementary Data 2, and the plasmids can be shared upon request.

### Generation of transgenic cassava lines

Genetic modification of the cassava cultivar TMS60444 was carried out as previously described^40^. *AKT2*_*var*_ and EV cassava lines were generated by transforming friable embryogenic calli with *Agrobacterium tumefaciens* containing either the binary vector p134GG_pAtAKT2::AtAKT2_var_, which carries the *AKT2*_*var*_ expression and the *hpt2* selectable marker cassettes, or the binary vector p134GG_Empty vector control, which contains only the *hpt2* selectable marker. The binary vector p134GG_pAtAKT2::GUS was transformed in the same way to generate the *p*At*AKT2*::*GUS* cassava lines. Hygromycin-resistant embryos were regenerated for all lines and screened to confirm the presence of the transgene.

### Plant tissue culture

Cassava EV control lines, *AKT2*_*var*_ lines and *p*At*AKT2*::*GUS* lines were grown in tissue culture containers (Greiner) on Murashige and Skoog medium at pH 5.8 (Murashige and Skoog basal salt mixture, including vitamins; Duchefa Biochemie) supplemented with 0.3% (w/v) Gelrite (Duchefa), 2% (v/v) sucrose and 2 µM CuSO_4_, under sterile conditions. The plants were grown under controlled conditions (16 h light/8 h dark; 100–120 μmol photons m^−2^ s^−1^, 28 °C in light/26 °C in dark) in a plant growth chamber before being transferred to soil for greenhouse and field trials.

### Plant culture in greenhouse experiments

Cassava EV control and *AKT2*_var_ transgenic lines were planted either from stem cuttings (stakes) or from sterile culture in standard soil (type ED73, Einheitserde Patzer) supplemented with 10% (v/v) sand. All plants were grown for 17 weeks in the greenhouse at RPTU Kaiserslautern, Germany (12 h light/12 h dark; 180 μmol photons m^−2^ s^−1^, 28 °C in light/26 °C in dark). Under standard conditions, the plants were watered regularly during the 17-week growth period.

### Drought stress growth conditions

For the drought stress experiments, plants were grown from cuttings and initially grown under standard conditions with a regular water supply for the first 8 weeks. Periodic drought stress was applied from week 9. Watering was stopped until the soil was noticeably dry and the plants showed phenotypic signs of drought stress, such as leaf wilting and subsequent abscission. After seven days of drought, the plants were watered with a fixed amount of water (100 ml). This watering schedule was repeated for 4 weeks to simulate periodic drought stress. After the 13th week of growth, the plants were again watered regularly to initiate recovery. The watering schedule is shown in Supplementary Fig. 9.

### Confined field trials

Confined field trials (CFTs) were conducted in 2022, 2023 and 2024 at the National Chung Hsing University Experimental Station in Taichung, Taiwan (latitude 24° 4′ 41.50″ N; longitude 120° 42′ 56.26″ E). The soil composition in the experimental field was a mixture of sand, silt and clay. Soil samples were taken regularly across the field to monitor the concentration and distribution of macronutrients (NH_4_^+^, NO_3_^−^, PO_4_^3−^, SO_4_^2−^ and K^+^), micronutrients (Mg, Na, Cu, Zn, Fe and Mn) and organic matter as well as the cation exchange capacity and pH. Cassava plants were imported from sterile tissue culture, transferred to soil in pots and then grown and hardened in a greenhouse for 2 months before being transferred to the field. After transfer, plants in the field were watered twice a week for 2 months or as necessary. The experimental field was reconditioned between CFTs using Taisugar No. 11 (10 t ha^−1^) and No. 4 ‘HeyWon’ nitrophosphate organic compound fertilizer (400 kg ha^−1^; Taiwan Fertilizer Company). The fields were ridged and covered with black tarpaulin to reduce weed growth. CFTs were conducted in a randomized serpentine design (Extended Data Fig. 3) with at least ten replicate plants for each independent transgenic event (line) at FH. During the growth season, cassava plants were treated as needed with a mixture of clofentezine and fenbutatin oxide (2.5 l ha^−1^) once a week for 2 to 3 weeks to control spider mites and thiamethoxam (500 g ha^−1^) to control scales. After a growth period of 8 to 9 months, the plants were harvested, and agronomic parameters such as plant height, SFW and RFW were recorded. DMC was determined by drying a representative piece of tissue. Total DMC was calculated by multiplying fresh weight by DMC. HI was calculated as root biomass divided by total biomass. Samples were collected during plant harvesting and processing as described below. The samples were freeze-dried, processed and sent to RPTU Kaiserslautern, Germany, for further analysis of ions, sugars and starch.

### Plant harvest and processing of greenhouse-grown plants

All greenhouse-grown plants were harvested after a total growth period of 17 weeks. At harvest, the height of each plant was measured before the plants were separated into three partitions: leaves, stems and storage roots. The weight of each partition was determined, and a tissue sample was collected and immediately frozen in liquid nitrogen for further analysis. When sampling the stem, part of the stem was separated into peel and core and frozen in liquid nitrogen for further processing. To prevent thawing of the samples, the frozen plant material was ground to a fine powder using a cryo mixer mill (Retsch MM400). A sample of 70 mg of the frozen plant material was taken for RNA isolation, and the fresh weight was measured using an analytical scale (Sartorius M-pact AX224). The plant material was then freeze-dried using a lyophilizer (Alpha 2-4 LDplus, Christ). After freeze-drying, the dry weight of the plant material was determined using an analytical scale, and samples of 10 mg each were used for analysis of ion, sugar and starch content.

### Southern blot analysis

Southern blot analysis was performed as previously described^41^ with the following specifications: 10 µg of genomic DNA was isolated^42^ and separated on a 1% agarose gel. After depurination, denaturation and neutralization, the DNA was blotted onto a nylon membrane and UV crosslinked. Hybridization was performed overnight in DIG easy Hyb buffer (Roche) with digoxigenin-labelled probes (PCR DIG labelling mix, Roche) directed against the *hpt2* coding sequence. After washing and blocking (blocking reagent, Roche), the probes were detected using an alkaline phosphatase-coupled anti-DIG antibody (Roche), CDP-star chemiluminescence substrate (Roche) and a ChemiDoc gel imaging system (Bio-Rad).

### RNA isolation

Frozen plant material from cassava leaves, stems (peel and core) and storage roots was used for RNA extraction. RNA was isolated using the STRN250-1KT Spectrum Plant Total RNA Kit (Sigma-Aldrich) according to the manufacturer’s specifications or by using the NucleoSpin RNA Plant Kit (Machery-Nagel). The purity and concentration of the extracted RNA were measured using a NanoDrop N60/N50 spectrophotometer (Implen) at a wavelength of 260 nm. For subsequent reverse transcription into complementary DNA (cDNA) using the qScript cDNA Synthesis Kit (Quantabio), 600 ng of RNA was used per reaction. Reverse transcription was performed using the Biometra Trio thermocycler (Analytik Jena) according to the manufacturer’s specifications. The incubation program used starts with reverse transcriptase activation at 25 °C for 5 min, followed by reverse transcription at 45 °C for 30 min. The reaction was terminated by heating the samples to 85 °C for 5 min. The resulting cDNA was cooled to 4 °C, diluted 1:6 with H_2_O and stored at −20 °C until use.

### RT–PCR analysis

RT–qPCR was performed using the Quantabio SYBR green quantification kit (Quantabio) on the PFX96 system (BioRad) using specific primers (Me*GAPDH* primer pair: TCTTCGGCGTTAGGAACCCAG/GCAGCCTTATCCTTGTCGGTG; At*AKT2* primer pair: ACAGGGGCTTAACGTCGACAC/TGCACCGTTAGTAGCCAGGAGA). The primers were checked for suitable amplification factor and primer efficiency (Me*GAPDH* 1.92/92.14%, At*AKT2* 2.07/106.59%). To quantify gene expression, the ΔCq value was calculated by subtracting the Cq value of the gene of interest from the Cq value of the housekeeping gene, Me*GAPDH*. RT–PCR was performed using specific primers (Me*GAPDH* primer pair: CGGCTTTTCCGGTATCCCTT/TCAAATGAGCGGCAGCCTTA; At*AKT2* primer pair: CAGCTTCTTGTCCGTGAACC/AGGTAAGCAGTGAGGCCAAG).

### GUS staining

Various cassava tissues were placed in ice-cold 90% acetone solution. Cross-sections were made manually with a razor blade. These sections were covered with GUS staining buffer (200 mM NaP pH7, 100 mM K_3_[Fe(CN_6_)], 100 mM K_4_[Fe(CN_6_)], 500 mM EDTA, 0.5% SILWET gold) and thoroughly vacuum infiltrated for 10 min. The GUS staining buffer was removed and replaced with fresh GUS staining solution containing GUS staining buffer with 0.25 mg ml^−1^ 5-bromo-4-chloro-3-indolyl-β-D-glucuronic acid (X-Gluc; pre-dissolved in 50 μl of DMSO). The GUS staining solution was thoroughly vacuum infiltrated for 10 min. The infiltrated tissues were incubated at 37 °C for 30 min. After removal of the GUS staining solution, 70% ethanol was added to the tissue sections and incubated at 37 °C until the tissues were clear. Images were captured using a Zeiss STEMI SV11 stereomicroscope (Zeiss).

### Quantification of ion concentrations

To isolate cations and anions, 800 µl of ddH_2_O was added to 10 mg of lyophilized plant material. The mixture was vortexed thoroughly using a Vortex-Genie 2 (Scientific Industries) and then incubated for 20 min at 95 °C and 500 rpm (Eppendorf Thermomixer Comfort). After incubation, the samples were again vortexed and placed on ice for 20 min to precipitate the starch. The plant material and starch were then pelleted via centrifugation at 16,000 *g* for 10 min at 4 °C in an Eppendorf centrifuge. 600 µl of the supernatant was transferred to a new reaction tube. Ion samples were diluted 1:5 in ddH_2_O for ion chromatography. Anion and cation concentrations were measured using a 761 Compact IC system (Metrohm). A Metrosep A Supp 4-250/4.0 column and a Metrosep A Supp 4/5 Guard/4.0 guard column (Metrohm) were used for the anion measurements. The eluent for anion measurements consisted of 1.8 mM Na_2_CO_3_ and 1.7 mM NaHCO_3_ dissolved in ultrapure water, with 50 mM H_2_SO_4_ as the anti-ion. A Metrosep C4 150/4.0 column and a Metrosep C4 Guard/4.0 guard column (Metrohm) were used for cation concentration measurements, with an eluent of 2 mM HNO_3_ and 1.6 mM dipicolinic acid dissolved in ultrapure water.

### Extraction of sugars and starch

Soluble metabolites, such as sugars, were extracted from 10 mg of lyophilized plant material. Sugars were extracted with 800 μl of 80% ethanol. After centrifugation at 16,000 *g* for 5 min, the supernatant was transferred to a new reaction tube, while the remaining pellet containing the plant material was retained for subsequent starch extraction. In preparation for measurement, the supernatant was evaporated using a Speedvac concentrator (Eppendorf). The resulting pellet was resuspended in 300 μl of ddH_2_O. Prior to starch extraction, the pellet from the sugar extraction was washed several times with 80% ethanol and water to remove any residual sugars. After the pellet was washed, 250 µl of ddH_2_O was added, and the samples were autoclaved at 121 °C for 20 min to hydrolyse the starch. For enzymatic starch digestion, 250 µl of a sodium acetate enzyme mastermix (containing 50 U ml^−1^ α-amylase, 6.3 U ml^−1^ amyloglucosidase and 200 mM NaOAc at pH 4.8) was added to the pellet and incubated at 37 °C for 4 h. The digestion was terminated by heating the samples to 95 °C for 10 min.

### Measurement of sugars and starch

Quantification of extracted sugars and hydrolysed starch was performed using an NADP^+^-coupled enzymatic assay as previously described^43^. For this analysis, the absorbance of NADPH was measured at a wavelength of 340 nm using a photometer (Tecan Infinite M Nano). The sugar concentration was then calculated according to the Lambert–Beer law.

### Measurement of amino acids

For the analysis of free amino acids, 20 µl of an 80% ethanol extract (800 μl) was mixed with 60 µl of borate buffer (200 mM, pH 8.8) and 20 µl of aminoquinolyl-*N*-hydroxysuccinimidyl carbamate (AQC) solution (Synchem UG & Co. KG; 2 mg ml^−1^ in acetonitrile). The mixture was immediately vortexed and incubated for 10 min to facilitate derivatization. Quantification of the derivatized AQC amino acids was performed using a Dionex P680 HPLC system with an RF 2000 fluorescence detector (Dionex) and a column system consisting of CC8/4 ND 100-5 C18ec and CC 250/4 ND 100-5 C18ec (Macherey-Nagel). A gradient of 100 mm sodium acetate/7 mm triethanolamine (pH 5.2, buffer A) and acetonitrile/water (90%, buffer B) (0–100%) was used for amino acid separation. The AQC-derivatized amino acids were detected fluorometrically with an excitation wavelength of 254 nm and an emission wavelength of 395 nm.

### ^11^C-PET analysis

Two days before and between the PET measurements, the plants were kept in a climate chamber at 28 °C, 65 ± 3% humidity and 400 ± 10 µmol m^−1^ s^−1^ photosynthetically active radiation at ambient CO_2_ concentration during the 16-h light period. During the 8-h dark period, the temperature was lowered to 22 ± 0.5 °C, and the humidity was kept constant. The climate chamber containing the PET instrument and the plant during the measurement was set to similar conditions.

On-site production of ^11^CO_2_ was achieved via the ^14^N(p,a)^11^C nuclear reaction by irradiation of N in a gas target with 18 MeV protons at the IBA 18/9 MeV cyclotron of the Institute of Plant Sciences ‘CYPRES’ at the Forschungszentrum Jülich GmbH (Germany). For transfer to the plant labelling circuit, the ^11^CO_2_ was collected in specially designed trapping devices as previously described^44^. At the end of the collection period, the activity in the closed trap was measured with a collimated scintillation detector (1″ NaI Scionix detector, Scionix) connected to an Osprey MCA (Mirion Technologies) before the trap was transferred to the labelling system. Plant labelling was performed as previously described^45^. Briefly, the activity in the labelling system was circulated in a closed loop until the target activity of 50 MBq was reached, at which point two valves were switched to include the plant leaf cuvette in the closed circuit for 6 min. After 6 min, the leaf cuvette was switched back to open mode. In open mode, the cuvette was again supplied with conditioned gas from a gas mixing unit, controlled at 26 ± 0.5 °C, 66 ± 4% humidity and 390 ± 10 ppm CO_2_, as before the measurement. The outflow from the cuvette was passed through a CO_2_ absorber encased in lead shielding to safely dispose of excess radioactivity. The inflow and outflow of the cuvette were monitored by the following sensors: a differential infrared gas analyser IRGA (LI-7000, LI-COR Biosciences GmbH), a mass flow meter (LowDeltaP, Bronkhorst Deutschland Nord GmbH), an atmospheric pressure sensor (144SC0811BARO, Sensortechnics, First Sensor) and a relative humidity and temperature sensor (AC3001, Rotronic Messgeräte GmbH). The resulting data were used to calculate the leaf assimilation rate as previously described^46^. Values are expressed as mean ± standard deviation for a 2-h period starting 5 min after the end of ^11^CO_2_ labelling. Leaf area was measured destructively after harvest (and used to calculate CO_2_ uptake per leaf area). The gas exchange measurement and labelling system is described elsewhere^47^. Labelling experiments were performed on the seventh or eighth leaf from the top of the plant, which was the youngest source leaf present on the plant.

The PET system used here, phenoPET, is a custom-built vertical bore instrument for plant measurements with a field of view 180 mm in diameter and 200 mm high. The details of the instrument and comparison with other plant-specific PET systems can be found elsewhere^48^. The system is mounted on a gantry so that it can be moved vertically around a potted plant, and the whole set-up is installed in a climate chamber. Images were reconstructed from the data using the PRESTO toolkit^49^.

In the reconstructed 3D images of the tracer signal distribution, cylindrical ROIs were placed along the stem (Fig. 3a). These ROIs serve as virtual detectors to capture the dynamics of tracer transport. The positions of the ROIs in the 3D PET image were determined using anatomical information from visual or imaging observations. The changing activity in these ROIs over time, resulting from ^11^C tracer that was assimilated by the plant after ^11^CO_2_ pulse labelling of a leaf and moving through the stem towards the root, was recorded as time-activity curves^50^. Tracer transport velocities were determined from the time-activity curves and the known spatial distances between ROIs using a compartmental transport model described in ref. ^51^. In the part of the stem directly below the petiole insertion (approximately 5–7 cm) connected to the ^11^CO_2_-labelled leaf, the data quality was suboptimal, so that only ROIs from the lower part of stem in the field of view were used for transport velocity analysis.

### Photosynthesis measurements

In 2022, photosynthesis data were collected from *AKT2*_*var*_ and EV plants in June at the CFT at the National Chung Hsing University Experimental Station, Taichung, Taiwan, using a mini-pulse amplitude modulation (PAM) system (MiniPAM; Heinz Walz). Measurements were taken on three top leaves under naturally fluctuating light conditions (Supplementary Fig. 13). ETR was calculated following ref. ^52^ and fitted to an exponential rise-to-maximum curve to derive ETR_max_ at saturation, indicating photosynthetic capacity^25^. In 2023 and 2024, photosynthesis data were collected in late November and mid-October, respectively, using a monitoring-PAM system (MoniPAM; Heinz Walz; Supplementary Fig. 13) configured to automatically record measurements every 15 min during daylight hours. ETR and ETR_max_ were computed as described above. Instrument examples are shown in Supplementary Fig. 13a,b, and measured plant locations for each year are shown in Supplementary Fig. 13c,e.

### Plant height measurements by UAV

UAV flight campaigns were carried out using an Okto-XL 6S12 microcopter (HiSystems GmbH). A high-resolution RGB camera (Sony Alpha 6000 with 35 mm lens; Sony Group Corporation) was used to collect images with 80% overlap (side and front) at 27 m above ground level, resulting in a pixel size of 0.003 m. Nadir images were collected near solar noon, typically between 11:00 and 13:00 local time, on a weekly or bi-weekly basis. A total of 30 ground control points were used to georeference the data on the basis of their known position measured by a real-time kinematic global navigation satellite system, achieving an accuracy of approximately 0.03 m. Individual raw images were further processed using the photogrammetric structure of the motion software Metashape (Agisoft LLC), from which georectified mosaic images and digital elevation models were generated. From the digital elevation model, the crop surface models were calculated, providing plant height information in metres above the ground level for each UAV data acquisition. Plant height per plant was calculated as the 95th quantile of the crop surface model values within an approximately 0.50-m buffer around each plant centre, aiming to reduce noise from outliers^53^. Plant volume was estimated as the sum of the crop surface model pixel values within each buffer, multiplied by the pixel area.

### Statistical analysis of field data

Data were processed in a stepwise manner to account for spatial and temporal variation. First, the data were corrected for field design and spatial trends by trait and year using the R package SpATS^24^ according to the formula:

$$Ys=f(r,c)+G+Rs+C$$

*Ys* represents the phenotypic value, *f*(*r*,*c*) is a smoothed bivariate surface defined over rows and columns, *G* is the genotype effect, *Rs* is the row effect and *C* is the column effect. The number of spline points was set to two thirds of the total number of rows and columns. On the basis of the spatial correction, outliers were excluded if the residual was more than three standard deviations from the mean.

The BLUEs and residual errors were retained as spatially corrected values^54,55^, which were further used for correlation and temporal analyses. In a second step, a linear mixed model was fitted to account for the temporal variation between the years. The previously spatially corrected values were used to calculate the genotypic BLUEs across the years for each trait using the R packages lme4 (ref. ^56^) and lmerTest^57^ according to the formula:

$$Yt=\mu +G+Y+Rt+\varepsilon$$

where *Yt* represents the spatially corrected value of the respective trait, *µ* is the overall mean, *G* is the fixed effect of the genotypes, *Y* is the random effect of years, *Rt* is the random effect of the replicates and *ε* is the residual error. Prior to modelling, an averaged EV control was calculated from the individual EV controls, which served as the reference group in the *t*-test.

### Reporting summary

Further information on research design is available in the Nature Portfolio Reporting Summary linked to this article.

## Supplementary information

Supplementary Information Supplementary Figs. 1–13 and uncropped scans for Extended Data Fig. 1b,c.

Reporting Summary

Supplementary Data 1 Source data for Supplementary Figs. 1–13.

Supplementary Data 2 Plasmids used in this study.

## Source data

Source Data Fig. 2 Statistical analysis and source data.

Source Data Fig. 3 Statistical analysis and source data.

Source Data Fig. 4 Statistical analysis and source data.

Source Data Fig. 5 Statistical analysis and source data.

Source Data Fig. 6 Statistical analysis and source data.

Source Data Extended Data Fig. 1 Uncropped blots and source data.

Source Data Extended Data Fig. 2 Statistical analysis and source data.

Source Data Extended Data Fig. 3 Statistical analysis and source data.

Source Data Extended Data Fig. 4 Statistical analysis and source data.

Source Data Extended Data Fig. 5 Statistical analysis and source data.

Source Data Extended Data Fig. 6 Statistical analysis and source data.

Source Data Extended Data Fig. 7 Statistical analysis and source data.

Source Data Extended Data Fig. 8 Statistical analysis and source data.

Source Data Extended Data Fig. 9 Statistical analysis and source data.

## Data Availability

Promoter and gene sequences of At*AKT2* (At4g22200) were used in this study. The sequence information can be found in Supplementary Data 2. Source data are provided with this paper.

## Code availability

All commands and scripts used in this study are available via GitHub at https://github.com/Division-of-Biochemistry-Publications/CASS.
