# Supplementary material for: Engineering vascular potassium transport increases yield and drought resilience of cassava
Source: Nat Plants. 2025 Dec 17;11(12):2498–510. doi: 10.1038/s41477-025-02159-7 (PMC12711561; doi:10.1038/s41477-025-02159-7)
Supplement: Supplementary file 1 — Supplementary Figs. 1–13 and uncropped scans for Extended Data Fig. 1b,c. [file 41477_2025_2159_MOESM1_ESM.pdf]

nature plants

Supplementary information https://doi.org/10.1038/s41477-025-02159-7

Engineering vascular potassium transport increases yield and drought resilience of cassava

In the format provided by the authors and unedited

Supplementary Fig. 1. Expression of *MeAKT2a* and *MeAKT2b* in cassava. (**A,B**) RNA expression profiles of *MeAKT2a* and *MeAKT2b*. DESeq2-normalized transcript data was published previously (Rüscher et al., 2024). PB = pre-bulking stage, approximately 30 days after planting (DAP), EB = early bulking stage, approximately 45 DAP, DB = during bulking stage, approximately 60 DAP. SiL = sink leaf, SoL = source leaf, Pet = petiole, US = upper stem, MS peel = middle stem peel, MS core = middle stem core, LS peel = lower stem peel, LS core = lower stem core, SR = storage root, FR = fibrous root. For PB n = 5,3,5,3,3,3,3,3,3,3, EB n = 5,5,5,5,5,5,5,4,5,5 and DB n = 5,3,5,4,4,4,4,4,4,4 biological replicates were analysed in A and B. (**C,D**) RNA expression profiles across the radial dimension of stem and storage root for *MeAKT2a* and *MeAKT2b*. DESeq2-normalized transcript data was published previously (Rüscher et al., 2025). From left to right n = 14,11,9,14,15,10,12,10 biological replicates were analysed in C and D. In box plots, centre line represents the median and plus (+) the mean, box edges delineate first and third quartiles, whiskers extend to maximum and minimum values and dots show individual values.

Supplementary Fig. 2. Expression of *AtAKT2*$_{var}$ in cassava causes only minor changes in cation and anion distribution in controlled greenhouse experiments. (**A**) Cation contents of potassium (K^+), calcium (Ca^{2+}), and magnesium (Mg^{2+}) in leaf tissue, lower stem, and storage root tissue. From left to right for EV-4218 n = 10,10,9,8,8,10,8,8,8, EV-4234 n = 10,10,9,8,8,10,8,8,8, EV-4243 n = 10,10,10,8,8,10,8,8,8, *AKT2*$_{var}$-4261 n = 5,5,5,4,5,5,5,4,5, *AKT2*$_{var}$-4262 n = 5,5,5,5,5,5,5,4,5 and *AKT2*$_{var}$-4264 n = 5,5,5,5,5,5,5,5,5 biological replicates were analysed. (**B**) Anion contents as phosphate (PO_4^{3-}), sulphate (SO_4^{2-}), and chloride (Cl^-) in leaf tissue, lower stem, and storage root tissue. From left to right for EV-4218 n = 11,13,13,11,13,14,12,13,13, EV-4234 n = 13,13,14,13,15,14,15,15,13, EV-4243 n = 13,14,12,13,14,14,14,14,13, *AKT2*$_{var}$-4261 n = 6,6,6,6,6,6,5,6,6, *AKT2*$_{var}$-4262 n = 6,5,5,6,6,5,5,6,5 and *AKT2*$_{var}$-4264 n = 6,6,6,6,6,6,6,6,6 biological replicates were analysed. Data in (**A**) and (**B**) are from plants of three EV control lines (EV-4218, EV-4234, and EV-4243) and three *AKT2*$_{var}$ lines (*AKT2*$_{var}$-4261, *AKT2*$_{var}$-4262, and *AKT2*$_{var}$-4264) 19 weeks after planting in soil. In box plots, centre line represents the median and plus (+) the mean, box edges delineate first and third quartiles, whiskers extend to maximum and minimum values and dots show individual values. Different lower-case letters indicate statistical significance, as calculated by one-way ANOVA with a post-hoc Tukey HSD test ($p < 0.05$).

Supplementary Fig. 3. *AtAKT2*$_{var}$ expression in cassava enhances shoot and root growth under controlled greenhouse conditions. Data are shown for three replicated experiments (Top to bottom = Cultivation I to III). (**A**) Phenotypes of shoots and roots of plants from EV control lines (EV-4234 and 4243), and plants from selected *AKT2*$_{var}$ lines (*AKT2*$_{var}$-4261, *AKT2*$_{var}$-4262, and *AKT2*$_{var}$-4264). (**B**) Shoot dry weight and (**C**) root dry weight was measured 19 weeks after planting in soil. In cultivation I for EV-4234 n = 8, EV-4243 n = 4, *AKT2*$_{var}$-4262 n = 4 and for *AKT2*$_{var}$-4264 n = 4 biological replicates were analysed. In cultivation II for EV-4234 n = 12, EV-4243 n = 10, *AKT2*$_{var}$-4262 n = 12 and for *AKT2*$_{var}$-4264 n = 12 biological replicates were analyzed. In cultivation III for EV-4234 n = 3, EV-4243 n = 3, *AKT2*$_{var}$-4261 n = 3, *AKT2*$_{var}$-4262 n = 7 and *AKT2*$_{var}$-4264 n = 5 biological replicates were analyzed. In box plots, centre line represents the median and plus (+) the mean, box edges delineate first and third quartiles, whiskers extend to maximum and minimum values and dots show individual values. Different lower-case letters indicate statistical significance, as calculated by one-way ANOVA with a post-hoc Tukey HSD test ($p < 0.05$).

Supplementary Fig. 4. Expression of *AKT2*$_{var}$ enhances cassava shoot and root growth under confined field trial conditions in 2022. (**A**) Stems and storage roots of representative plants from EV control and *AKT2*$_{var}$ lines. (**B**) Shoot fresh weight, (**C**) root fresh weight, and (**D**) dry matter content was quantified about 9 months (April to December) after planting in 2022. For all lines n = 10 biological replicates were analyzed per line. In box plots, centre line represents the median and plus (+) the mean, box edges delineate first and third quartiles, whiskers extend to maximum and minimum values and dots show individual values. Different lower-case letters indicate statistical significance, as calculated by one-way ANOVA with a post-hoc Tukey HSD test ($p < 0.05$). Scale bars are 20 cm.

Supplementary Fig. 5. Shoot and root growth of plants from *AKT2$_{var}$* and EV cassava lines under confined field trial conditions in 2023. (**A**) Stems and storage roots of representative plants from EV control and *AKT2$_{var}$* lines. (**B**) Shoot fresh weight, (**C**) root fresh weight, and (**D**) dry matter content was quantified about 9 months (April to December) after planting in 2023. For EV-4218 n = 17, EV-4220 n = 18, EV-4221 n = 18, EV-4234 n = 18, EV-4243 n = 18, *AKT2$_{var}$*-4255 n = 17, *AKT2$_{var}$*-4261 n = 17, *AKT2$_{var}$*-4262 n = 17, *AKT2$_{var}$*-4265 n = 17 and *AKT2$_{var}$*-4266 n = 18 biological replicates were analysed. In box plots, centre line represents the median and plus (+) the mean, box edges delineate first and third quartiles, whiskers extend to maximum and minimum values and dots show individual values. Different lower-case letters indicate statistical significance, as calculated by one-way ANOVA with a post-hoc Tukey HSD test ($p < 0.05$). Scale bars are 20 cm.

Supplementary Fig. 6. Shoot and root growth of plants from *AKT2$_{var}$* and EV cassava lines under confined field trial conditions in 2024. (**A**) Stems and storage roots of representative plants from EV and *AKT2$_{var}$* lines. (**B**) Shoot fresh weight, (**C**) root fresh weight, and (**D**) dry matter content was quantified about 9 months (April to December) after planting in 2024. For EV-4218 n = 10, EV-4220 n = 11, EV-4221 n = 10, EV-4234 n = 10, EV-4243 n = 11, *AKT2$_{var}$*-4255 n = 12, *AKT2$_{var}$*-4261 n = 12, *AKT2$_{var}$*-4262 n = 10, *AKT2$_{var}$*-4265 n = 21 and *AKT2$_{var}$*-4266 n = 11 biological replicates were analysed. In box plots, centre line represents the median and plus (+) the mean, box edges delineate first and third quartiles, whiskers extend to maximum and minimum values and dots show individual values. Different lower-case letters indicate statistical significance, as calculated by one-way ANOVA with a post-hoc Tukey HSD test ($p < 0.05$). Scale bars are 20 cm.

Supplementary Fig. 7. Summary of UAV-phenotyping data recorded during confined field trials. Comparisons between plants from five EV and five *AKT2$_{var}$* lines grown during confined field trials in 2022 to 2024. At least 10 biological replicates were analyzed per line and year. (**A**) Time course of plant height calculated from UAV data. (**B**) Time course of plant canopy volume calculated from UAV data. UAV = Unmanned aerial vehicle.

Supplementary Fig. 8. Weather data showing differences in rainfall during the 2022, 2023, and 2024 confined field trials. Precipitation data and the temperature measurements from field trials in (**A**) 2022, (**B**) 2023, and (**C**) 2024. Harvests were completed about nine months after transfer of cassava plantlets to the field.

Supplementary Fig. 9. Schematic representation of the cultivation process to induce drought stress in cassava under greenhouse conditions. To induce drought stress in the EV and *AKT2$_{var}$* lines, all plants were first grown under controlled watered conditions for 8 weeks. This was followed by a five-week drought stress period during which half of the plants were watered only once a week, while control plants were continued to be watered daily. An intermediate harvest was carried out after five weeks of drought, after which all plants were again watered daily. The final harvest was carried out after a further five weeks of growth.

Supplementary Fig. 10. *AKT2$_{var}$* expression is increased in response to drought stress. Quantitative real-time PCR measurements of relative *AKT2$_{var}$* mRNA expression levels in the lower stem of empty vector control (EV-4243) and *AKT2*$_{var}$ lines (*AKT2*$_{var}$-4261 and *AKT2*$_{var}$-4262) after normalization to *MeGAPDH* under standard conditions (STD) and drought stress (DS). For EV-4243 n = 4, *AKT2*$_{var}$-4261 n = 5 and *AKT2*$_{var}$-4262 n = 4,5 biological replicates were analyzed. In box plots, centre line represents the median and plus (+) the mean, box edges delineate first and third quartiles, whiskers extend to maximum and minimum values and dots show individual values. For statistical significance students t-test were performed (p < 0.05 **; p < 0.01 ***).

Supplementary Fig. 11. *AKT2$_{var}$* expression in cassava does not cause changes in cation distribution during periodic drought stress. Concentrations of potassium (K^+), calcium (Ca^{2+}), and magnesium (Mg^{2+}) in leaf, lower stem, and fibrous root tissues of plants from EV lines (EV-4221, EV-4234, and EV-4243) and *AKT2$_{var}$* lines (*AKT2$_{var}$*-4261, *AKT2$_{var}$*-4262, and *AKT2$_{var}$*-4264) under (**A**) control conditions or (**B**) during periodic drought stress. In A and B for EV-4221 n = 7,7,7,7,7,7,6,6,6, EV-4243 n = 6,6,6,6,6,6,6,6,6, EV-4243 n = 7,7,7,6,6,6,7,7,7, *AKT2*$_{var}$-4261 n = 6,6,6,6,6,6,6,6,6, *AKT2*$_{var}$-4262 n = 5,5,5,5,5,5,5,5,5, and *AKT2*$_{var}$-4264 n = 7,7,7,7,7,7,7,7,7 biological replicates were analyzed. In box plots, centre line represents the median and plus (+) the mean, box edges delineate first and third quartiles, whiskers extend to maximum and minimum values and dots show individual values. Different lower-case letters indicate statistical significance, as calculated by one-way ANOVA with a post-hoc Tukey HSD test ($p < 0.05$).

Supplementary Fig. 12. *AKT2$_{var}$* expression in cassava causes only minor changes in anion distributions during periodic drought stress. Shown are cation contents of phosphate (PO_4^{3-}), sulphate (SO_4^{2-}), and chloride (Cl^-) in leaf, lower stem, and fibrous root tissues of plants from EV lines (EV-4221, 4234, and 4243) and *AKT2$_{var}$* lines (*AKT2$_{var}$*-4261, 4262, and 4264) under (**A**) control conditions or (**B**) during periodic drought stress. In A for EV-4221 n = 6,6,6,7,7,7,7,7,5, EV-4243 n = 6,6,6,6,6,6,6,6,6, EV-4243 n = 7,7,7,7,7,7,7,7,5 *AKT2*$_{var}$-4261 n = 6,6,6,6,6,6,6,6,5 *AKT2*$_{var}$-4262 n = 5,5,5,5,5,5,5,5,5, and *AKT2*$_{var}$-4264 n = 7,7,7,7,7,7,7,7,6 biological replicates were analyzed. In B for EV-4221 n = 7,7,7,7,7,7,7,7,7, EV-4243 n = 6,6,6,6,6,6,6,6,6, EV-4243 n = 7,7,7,7,7,7,6,6,6, *AKT2*$_{var}$-4261 n = 6,6,6,6,6,6,6,6,6 *AKT2*$_{var}$-4262 n = 5,5,5,5,5,5,4,4,4, and *AKT2*$_{var}$-4264 n = 7,7,7,7,7,7,7,7,7 biological replicates were analyzed. In box plots, centre line represents the median and plus (+) the mean, box edges delineate first and third quartiles, whiskers extend to maximum and minimum values and dots show individual values. Different lower-case letters indicate statistical significance, as calculated by one-way ANOVA with a post-hoc Tukey HSD test ($p < 0.05$).

Supplementary Fig. 13. Photosynthetic phenotyping and elevation mapping of cassava genotypes across three seasons. (**A**) Mini-PAM fluorometry setup used in the 2022 field campaign. (**B**) Moni-PAM system applied in the 2023/24 season for high-throughput diurnal measurements. (**C-E**) UAV-derived elevation maps of selected field blocks acquired on July 7, 2022 (2022), July 25, 2023 (2023), and August 28, 2024 (2024). The colour scale represents relative surface elevation (0.0–2.5 m), derived from canopy surface models. Plants used for photosynthesis are indicated by red markers belonging to the *AKT2$_{var}$* genotype group, while grey markers indicate EV (empty vector) control lines.

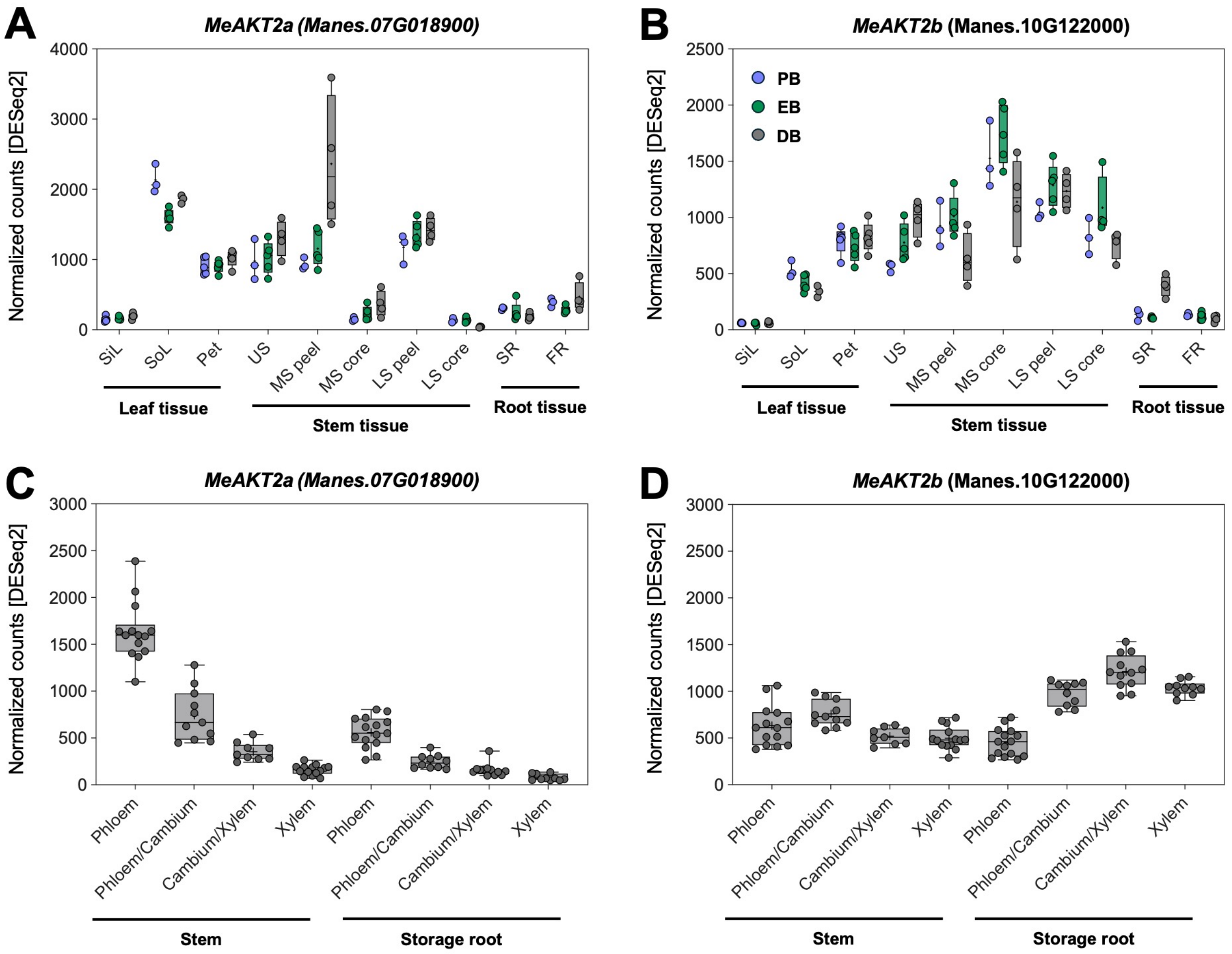

Supplementary Fig. 1. Expression of *MeAKT2a* and *MeAKT2b* in cassava.

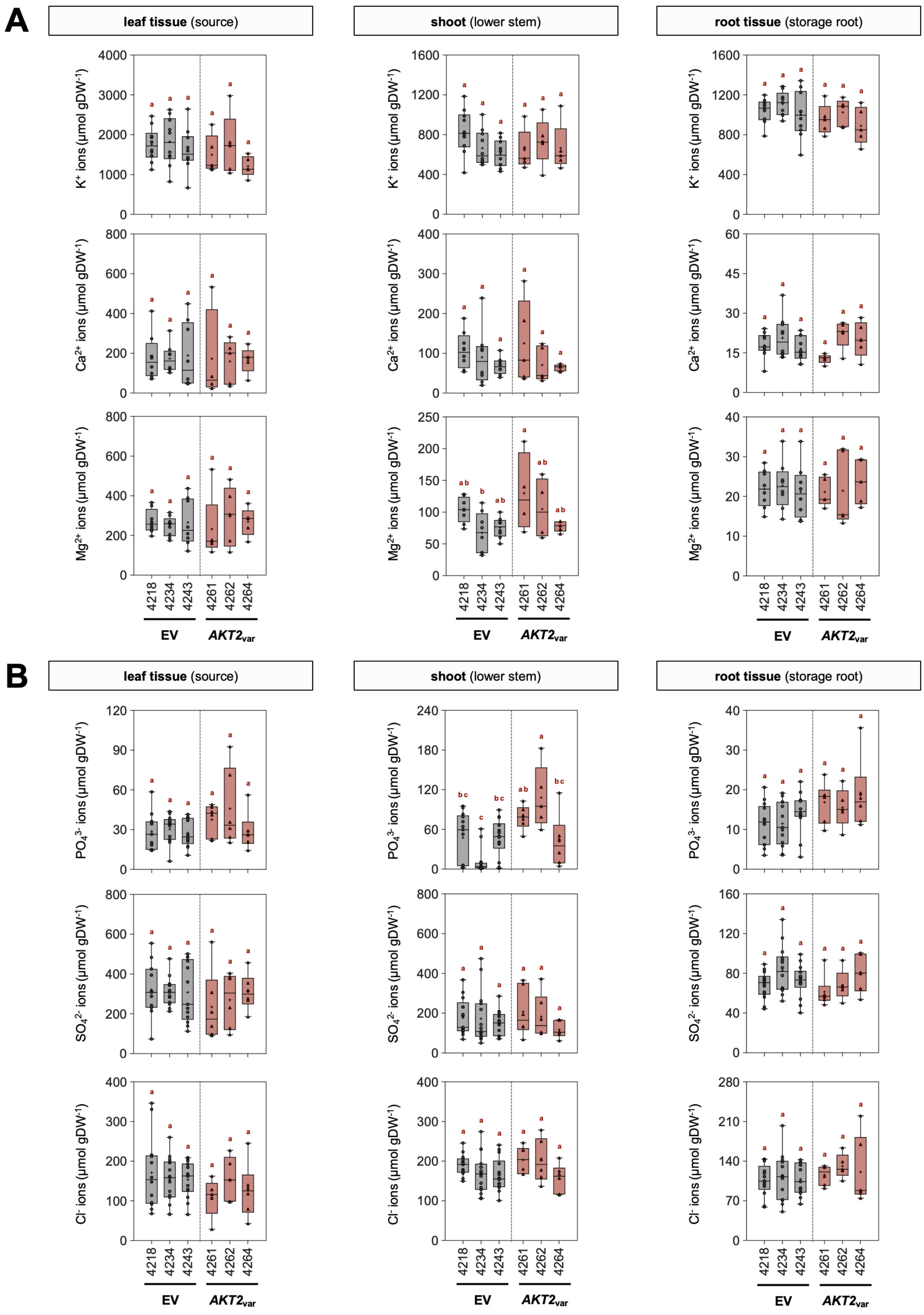

Supplementary Fig. 2. Expression of *AtAKT2$_{var}$* in cassava causes only minor changes in cation and anion distribution in controlled greenhouse experiments.

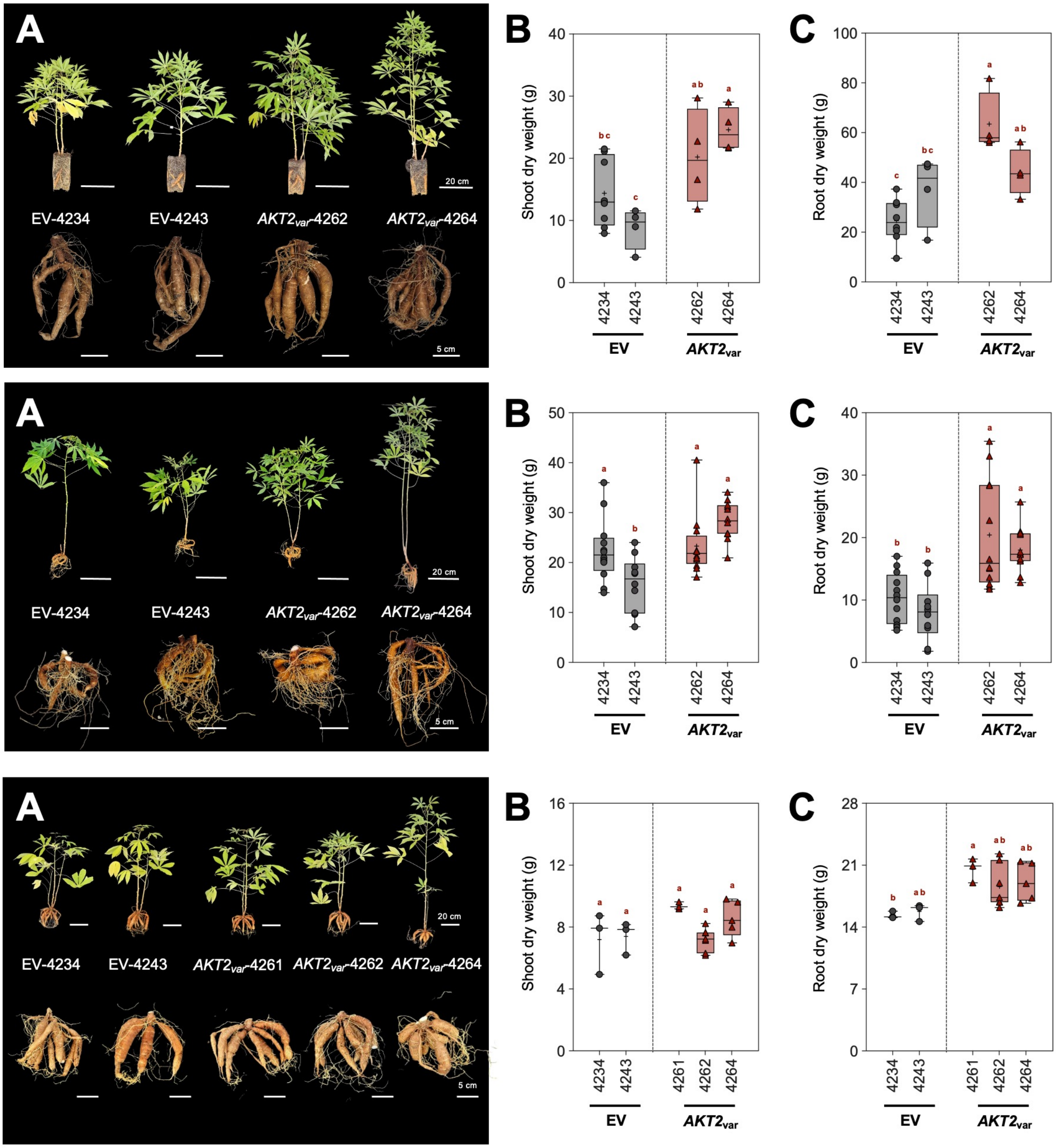

Supplementary Fig. 3. *AtAKT2$_{var}$* expression in cassava enhances shoot and root growth under controlled greenhouse conditions.

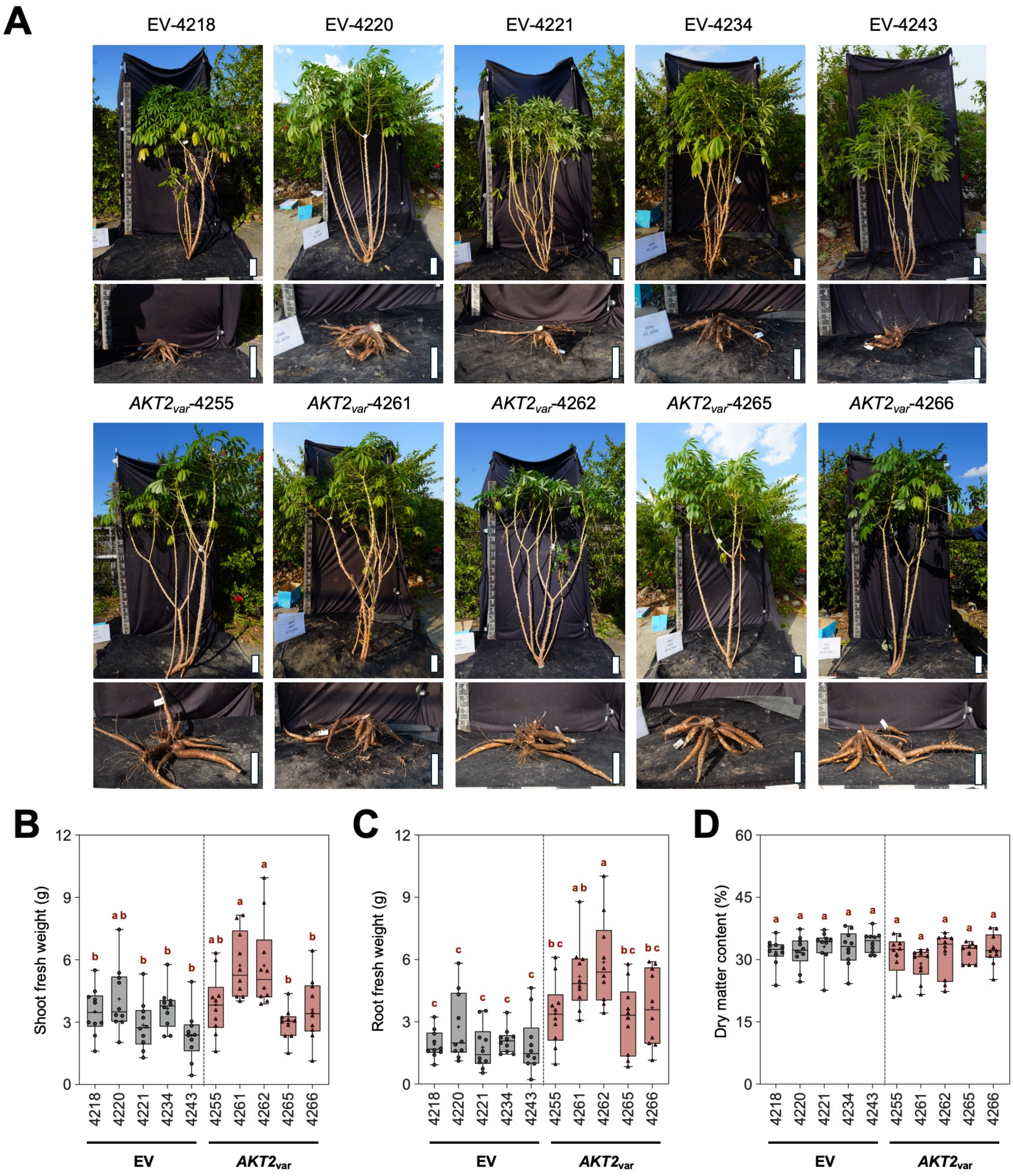

Supplementary Fig. 4. Expression of *AKT2*$_{var}$ enhances cassava shoot and root growth under confined field trial conditions in 2022.

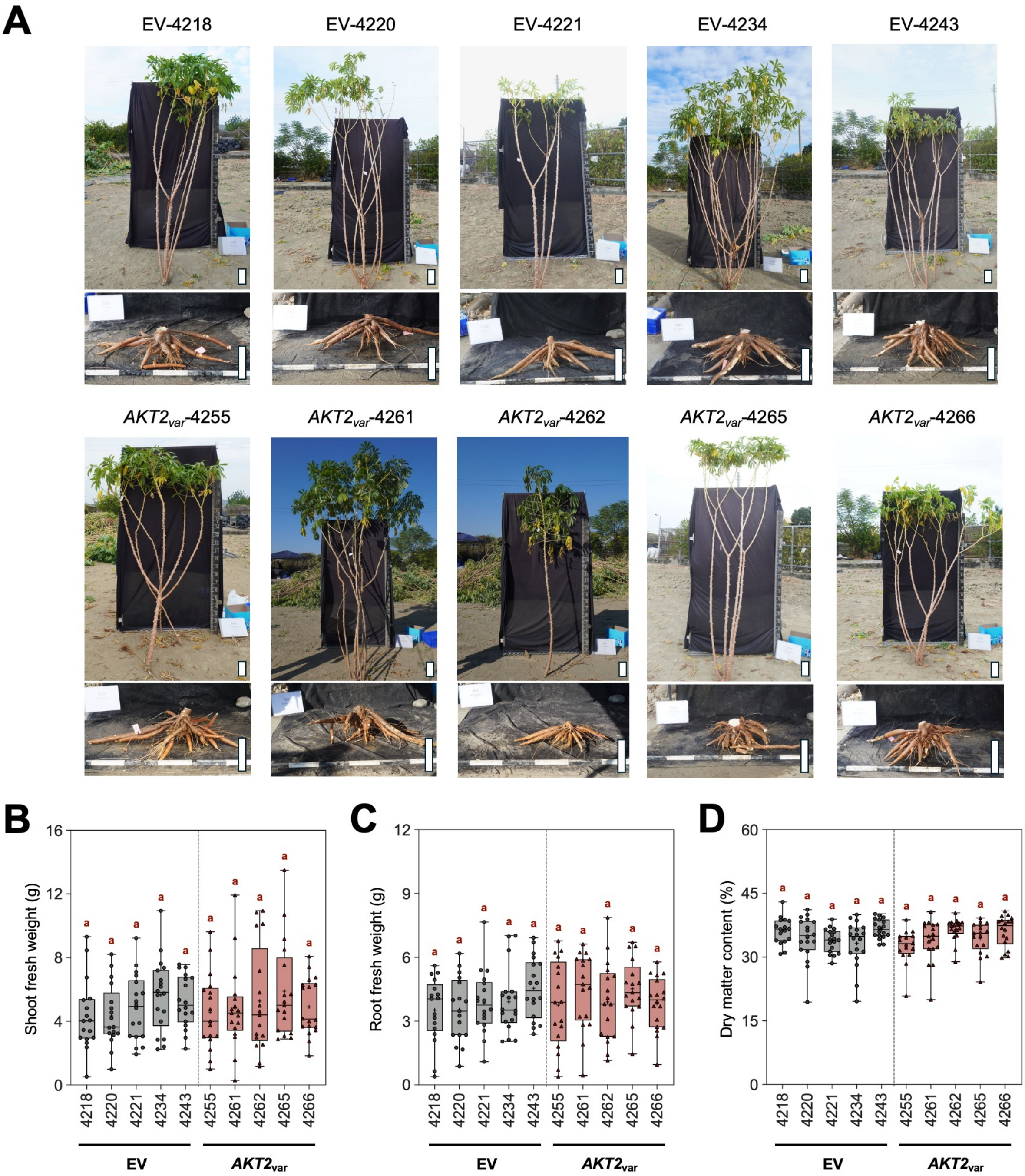

Supplementary Fig. 5. Shoot and root growth of plants from *AKT2$_{var}$* and EV cassava lines under confined field trial conditions in 2023..

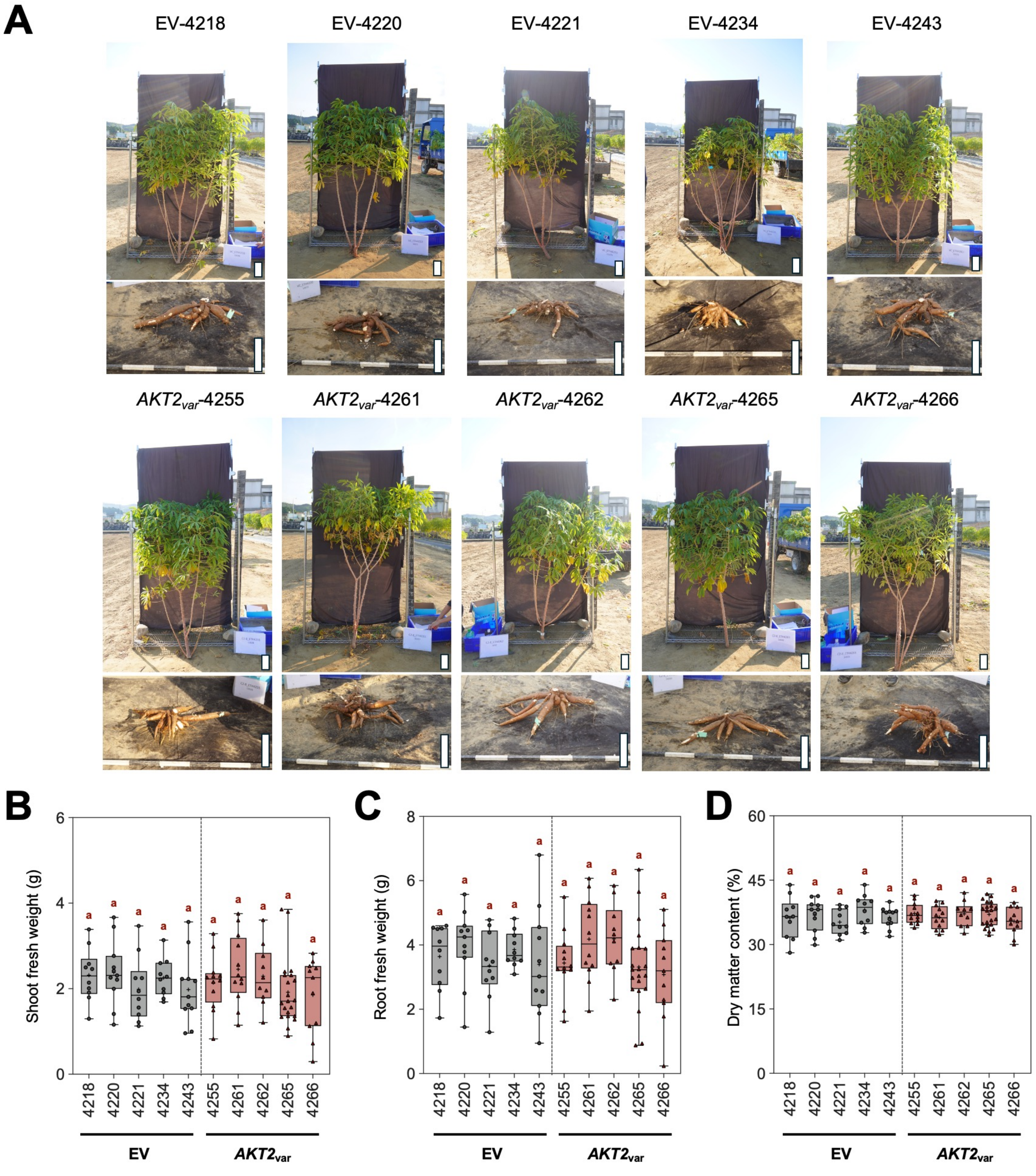

Supplementary Fig. 6. Shoot and root growth of plants from *AKT2$_{var}$* and EV cassava lines under confined field trial conditions in 2024.

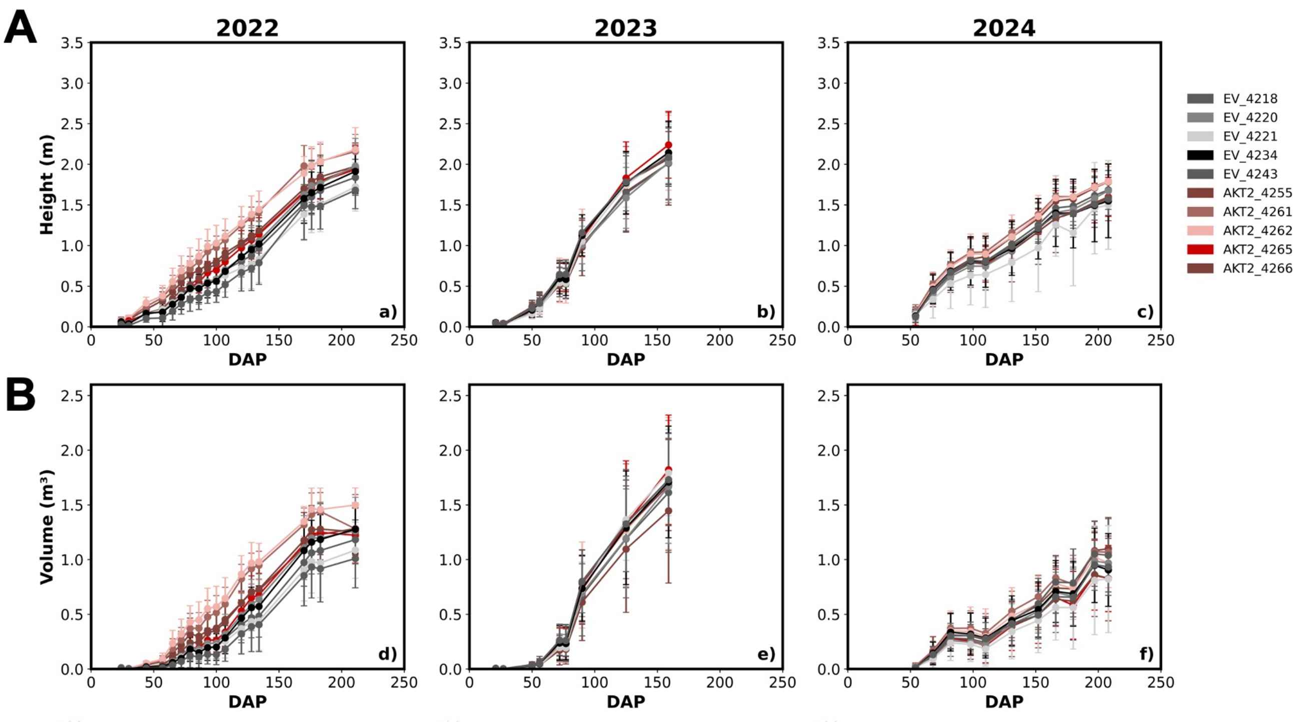

Supplementary Fig. 7. Summary of UAV-phenotyping data recorded during confined field trials.

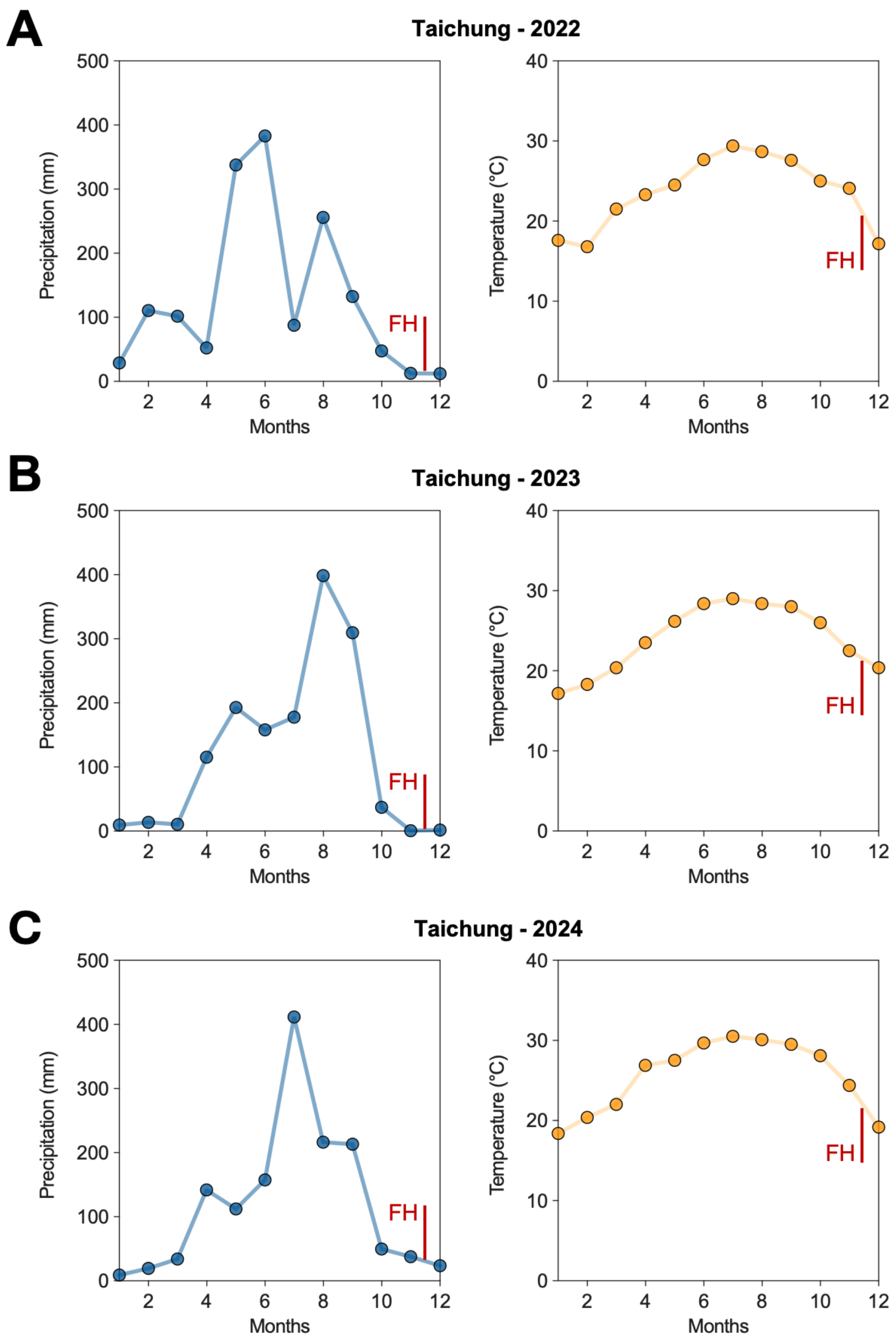

Supplementary Fig. 8. Weather data showing differences in rainfall during the 2022, 2023, and 2024 confined field trials.

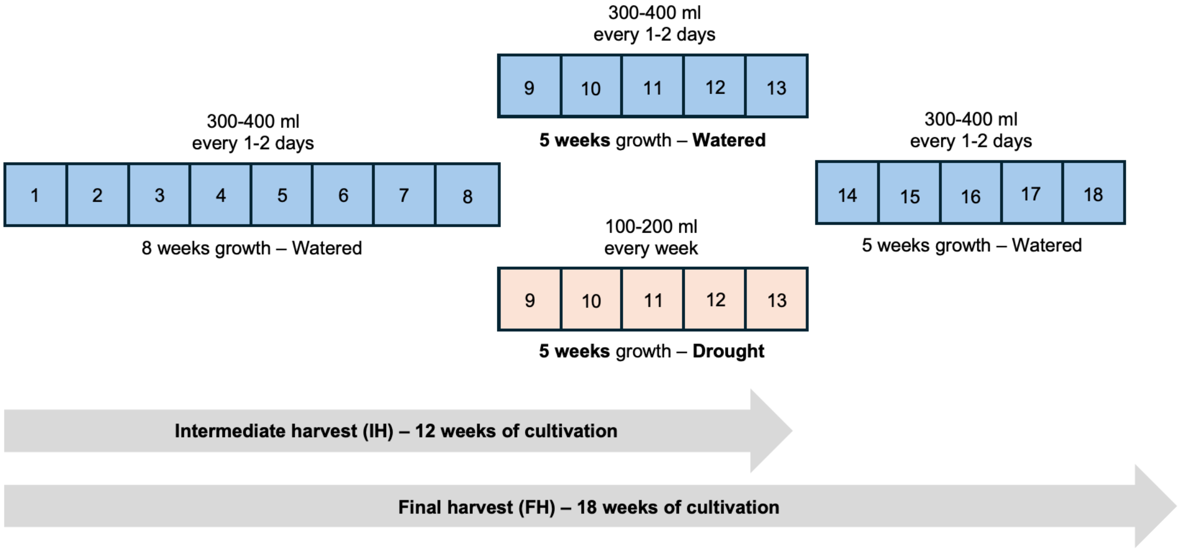

Supplementary Fig. 9. Schematic representation of the cultivation process to induce drought stress in cassava under greenhouse conditions.

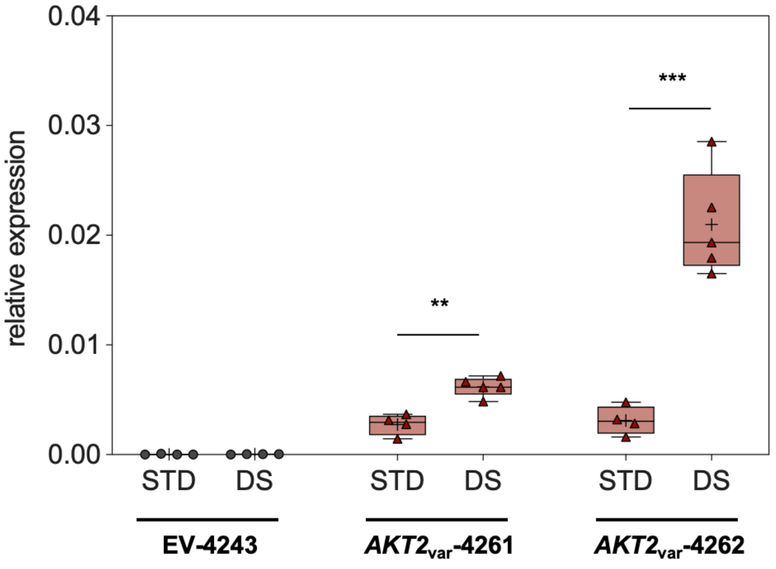

Supplementary Fig. 10. *AKT2$_{var}$* expression is increased in response to drought stress.

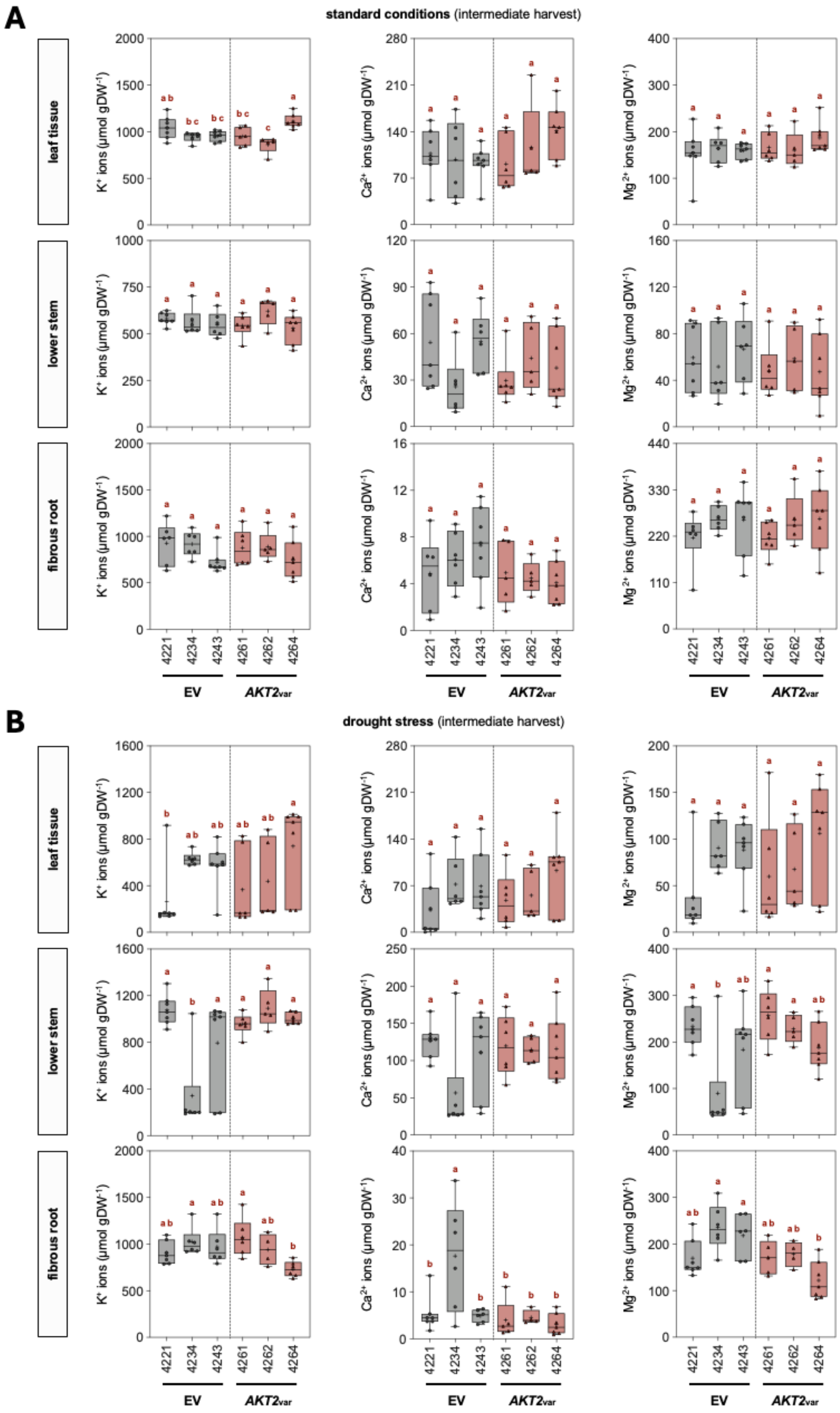

Supplementary Fig. 11. ***AKT2$_{var}$*** **expression in cassava does not cause changes in cation distribution during periodic drought stress.**

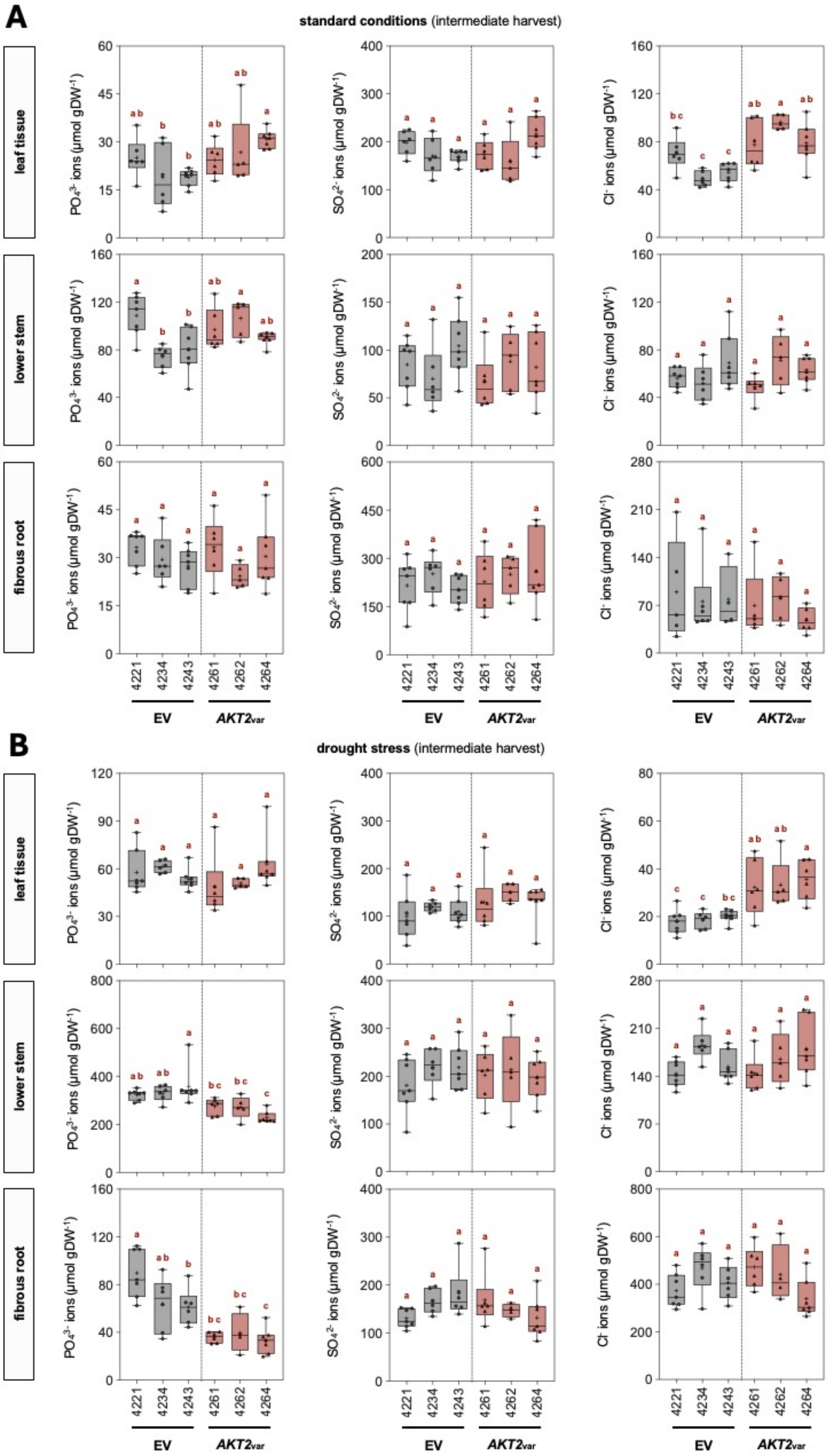

Supplementary Fig. 12. ***AKT2****$_{var}$* **expression in cassava causes only minor changes in anion distributions during periodic drought stress.**

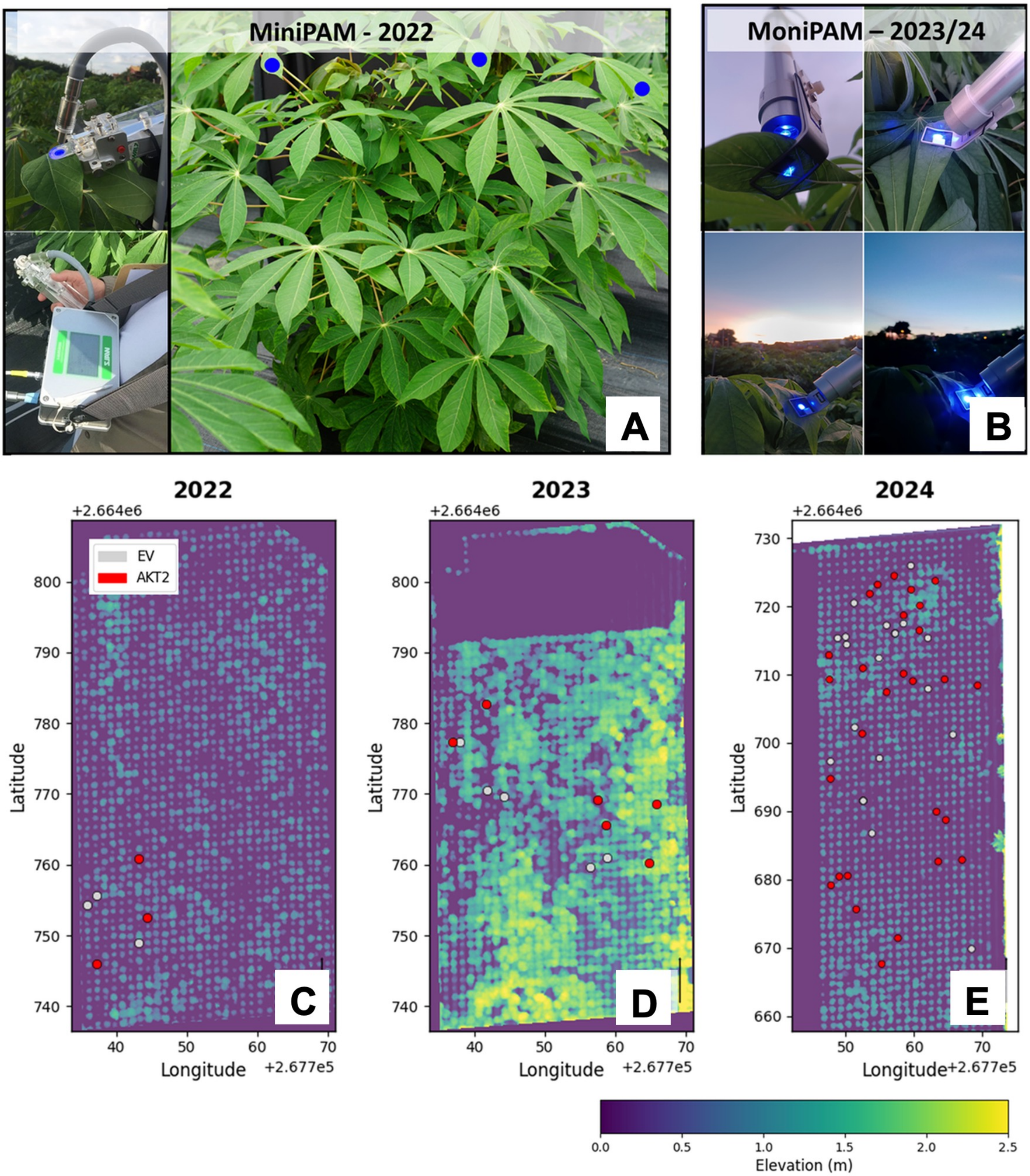

Supplementary Fig. 13. Photosynthetic phenotyping and elevation mapping of cassava genotypes across three seasons.
