# Supplementary material for: Engineering vascular potassium transport increases yield and drought resilience of cassava
Source: Nat Plants. 2025 Dec 17;11(12):2498–510. doi: 10.1038/s41477-025-02159-7 (PMC12711561; doi:10.1038/s41477-025-02159-7)
Supplement: Supplementary file 2 — Reporting Summary [file 41477_2025_2159_MOESM2_ESM.pdf]

nature portfolio

Corresponding author(s): Wolfgang Zierer, Wilhelm Gruissem, Leo Bellin

Last updated by author(s): Oct 7, 2025

Reporting Summary

Nature Portfolio wishes to improve the reproducibility of the work that we publish. This form provides structure for consistency and transparency in reporting. For further information on Nature Portfolio policies, see our Editorial Policies and the Editorial Policy Checklist.

Statistics

For all statistical analyses, confirm that the following items are present in the figure legend, table legend, main text, or Methods section.

n/a	Confirmed	
☐	☒	The exact sample size (*n*) for each experimental group/condition, given as a discrete number and unit of measurement
☐	☒	A statement on whether measurements were taken from distinct samples or whether the same sample was measured repeatedly
☐	☒	The statistical test(s) used AND whether they are one- or two-sided<br>*Only common tests should be described solely by name; describe more complex techniques in the Methods section.*
☐	☒	A description of all covariates tested
☐	☒	A description of any assumptions or corrections, such as tests of normality and adjustment for multiple comparisons
☐	☒	A full description of the statistical parameters including central tendency (e.g. means) or other basic estimates (e.g. regression coefficient) AND variation (e.g. standard deviation) or associated estimates of uncertainty (e.g. confidence intervals)
☐	☒	For null hypothesis testing, the test statistic (e.g. *F*, *t*, *r*) with confidence intervals, effect sizes, degrees of freedom and *P* value noted<br>*Give P values as exact values whenever suitable.*
☒	☐	For Bayesian analysis, information on the choice of priors and Markov chain Monte Carlo settings
☒	☐	For hierarchical and complex designs, identification of the appropriate level for tests and full reporting of outcomes
☒	☐	Estimates of effect sizes (e.g. Cohen's *d*, Pearson's *r*), indicating how they were calculated

Our web collection on statistics for biologists contains articles on many of the points above.

Software and code

Policy information about availability of computer code

Data collection	Data collection was performed as described in the material and methods section.
Data analysis	Data analysis was performed as described in the material and methods section. Field data and UAV data was evaluated using R. All used commands and scripts are available on GitHub: https://github.com/Division-of-Biochemistry-Publications/CASS. All other data was evaluated using GraphPad Prism Version 10

For manuscripts utilizing custom algorithms or software that are central to the research but not yet described in published literature, software must be made available to editors and reviewers. We strongly encourage code deposition in a community repository (e.g. GitHub). See the Nature Portfolio guidelines for submitting code & software for further information.

Data

Policy information about availability of data

All manuscripts must include a data availability statement. This statement should provide the following information, where applicable:

- Accession codes, unique identifiers, or web links for publicly available datasets
- A description of any restrictions on data availability
- For clinical datasets or third party data, please ensure that the statement adheres to our policy

All data used in this study is available in the source data files. Promoter and gene sequences of AtAKT2 (At4g22200) were used in this study. Sequence information can be found in Supplementary Data S2.

Research involving human participants, their data, or biological material

Policy information about studies with human participants or human data. See also policy information about sex, gender (identity/presentation), and sexual orientation and race, ethnicity and racism.

Reporting on sex and gender	NA
Reporting on race, ethnicity, or other socially relevant groupings	NA
Population characteristics	NA
Recruitment	NA
Ethics oversight	NA

Note that full information on the approval of the study protocol must also be provided in the manuscript.

Field-specific reporting

Please select the one below that is the best fit for your research. If you are not sure, read the appropriate sections before making your selection.

☒ Life sciences ☐ Behavioural & social sciences ☐ Ecological, evolutionary & environmental sciences

For a reference copy of the document with all sections, see nature.com/documents/nr-reporting-summary-flat.pdf

Life sciences study design

All studies must disclose on these points even when the disclosure is negative.

Sample size	No sample size calculations were performed. We aimed to use as many biological replicates per plant line as possible, however, cassava plants are comparatively large and can quickly cause size restrictions in greenhouse and field trials. Typically, at least 4 biological replicates were used in greenhouse trials, and at least 10 biological replicates were used in field trials.
Data exclusions	No data were excluded where measurable values occurred in the analyses. Only 0 values or if no detectable values were quantifiable were removed.
Replication	Greenhouse experiments under standard conditions were repeated at least four times. Greenhouse experiments under drought conditions were repeated three times in independent cultivations including C1 (EV-4221, AKT2var-4261 and AKT2var-4262), C2 (EV-4221, EV-4234, EV-4343, AKT2var-4261, AKT2var-4262 and AKT2var-4264) and C3 (EV-4234, AKT2var-4261 and AKT2var-4262). Three independent field trials from 2022-2024 were performed.
Randomization	Plants were fully randomized in greenhouse trials. Field trials were conducted according to a randomized serpentine design.
Blinding	No blinding

Reporting for specific materials, systems and methods

We require information from authors about some types of materials, experimental systems and methods used in many studies. Here, indicate whether each material, system or method listed is relevant to your study. If you are not sure if a list item applies to your research, read the appropriate section before selecting a response.

Materials & experimental systems

n/a	Involved in the study	
☒	☐	Antibodies
☒	☐	Eukaryotic cell lines
☒	☐	Palaeontology and archaeology
☒	☐	Animals and other organisms
☒	☐	Clinical data
☒	☐	Dual use research of concern
☐	☒	Plants

Methods

n/a	Involved in the study	
☒	☐	ChIP-seq
☒	☐	Flow cytometry
☒	☐	MRI-based neuroimaging

Dual use research of concern

Policy information about dual use research of concern

Hazards

Could the accidental, deliberate or reckless misuse of agents or technologies generated in the work, or the application of information presented in the manuscript, pose a threat to:

No	Yes	
☒	☐	Public health
☒	☐	National security
☒	☐	Crops and/or livestock
☒	☐	Ecosystems
☒	☐	Any other significant area

Experiments of concern

Does the work involve any of these experiments of concern:

No	Yes	
☒	☐	Demonstrate how to render a vaccine ineffective
☒	☐	Confer resistance to therapeutically useful antibiotics or antiviral agents
☒	☐	Enhance the virulence of a pathogen or render a nonpathogen virulent
☒	☐	Increase transmissibility of a pathogen
☒	☐	Alter the host range of a pathogen
☒	☐	Enable evasion of diagnostic/detection modalities
☒	☐	Enable the weaponization of a biological agent or toxin
☒	☐	Any other potentially harmful combination of experiments and agents

Plants

Seed stocks	TMS60444 WT plants were initially received from the International Institute of Tropical Agriculture (IITA) Ibadan by Prof. Wilhelm Gruissem at ETH Zurich (Division of Plant Biotechnology) and subsequently gifted to Prof. Uwe Sonnewald at FAU Erlangen.
Novel plant genotypes	Cloning and transformation is described in detail in the Material & Methods section. Both the pAtAKT2::GUS and the pAtAKT2::ATK2var transgenic cassava lines were produced by the transformation method of Bull et al., 2009.
Authentication	pAtAKT2::AtAKT2var lines: Transgenic plant lines were tested by PCR for the presence of the T-DNA in the plant genome. T-DNA insertion events were determined by Southern Blot analysis. Transgene expression was confirmed by qRT-PCR. Three independent lines have been used for the greenhouse experiments and five independent lines have been used for the field experiments. pAtAKT2::GUS lines: Transgenic plant lines were tested by PCR for the presence of the T-DNA in the plant genome. Four independent lines have been analyzed by detailed GUS staining.
